# Loss of E-cadherin leads to Id2-dependent inhibition of cell cycle progression in metastatic lobular breast cancer

**DOI:** 10.1038/s41388-022-02314-w

**Published:** 2022-04-18

**Authors:** Max A. K. Rätze, Thijs Koorman, Thijmen Sijnesael, Blessing Bassey-Archibong, Robert van de Ven, Lotte Enserink, Daan Visser, Sridevi Jaksani, Ignacio Viciano, Elvira R. M. Bakker, François Richard, Andrew Tutt, Lynda O’Leary, Amanda Fitzpatrick, Pere Roca-Cusachs, Paul J. van Diest, Christine Desmedt, Juliet M. Daniel, Clare M. Isacke, Patrick W. B. Derksen

**Affiliations:** 1 Department of Pathology, University Medical Center Utrecht, Utrecht, The Netherlands; 2 Department of Biology, McMaster University, Hamilton, ON Canada; 3 Institute for Bioengineering of Catalonia (IBEC), the Barcelona Institute of Technology (BIST), Barcelona, Spain; 4 Laboratory for Translational Breast Cancer Research, Katholieke Universiteit, Leuven, Belgium; 5 The Breast Cancer Now Research Unit, King’s College London, London, United Kingdom; 6 Breast Cancer Now Toby Robins Research Centre, The Institute of Cancer Research, London, United Kingdom

**Keywords:** Breast cancer, Cadherins

## Abstract

Invasive lobular breast carcinoma (ILC) is characterized by proliferative indolence and long-term latency relapses. This study aimed to identify how disseminating ILC cells control the balance between quiescence and cell cycle re-entry. In the absence of anchorage, ILC cells undergo a sustained cell cycle arrest in G0/G1 while maintaining viability. From the genes that are upregulated in anchorage independent ILC cells, we selected Inhibitor of DNA binding 2 (*Id2*), a mediator of cell cycle progression. Using loss-of-function experiments, we demonstrate that Id2 is essential for anchorage independent survival (anoikis resistance) in vitro and lung colonization in mice. Importantly, we find that under anchorage independent conditions, E-cadherin loss promotes expression of Id2 in multiple mouse and (organotypic) human models of ILC, an event that is caused by a direct p120-catenin/Kaiso-dependent transcriptional de-repression of the canonical Kaiso binding sequence *TCCTGCNA*. Conversely, stable inducible restoration of E-cadherin expression in the ILC cell line SUM44PE inhibits Id2 expression and anoikis resistance. We show evidence that Id2 accumulates in the cytosol, where it induces a sustained and CDK4/6-dependent G0/G1 cell cycle arrest through interaction with hypo-phosphorylated Rb. Finally, we find that Id2 is indeed enriched in ILC when compared to other breast cancers, and confirm cytosolic Id2 protein expression in primary ILC samples. In sum, we have linked mutational inactivation of E-cadherin to direct inhibition of cell cycle progression. Our work indicates that loss of E-cadherin and subsequent expression of Id2 drive indolence and dissemination of ILC. As such, E-cadherin and Id2 are promising candidates to stratify low and intermediate grade invasive breast cancers for the use of clinical cell cycle intervention drugs.

## Introduction

Clinical outcome for invasive lobular breast cancer (ILC) patients with metastatic disease is dismal once tumors stop responding to estrogen receptor antagonists [1]. ILC has a propensity to be chemo-refractory, a feature that underpins the unsatisfactory clinical responses to standard (neo-adjuvant) treatments [1, 2]. ILC development and progression are causally related to loss of cell-cell adhesion through inactivation of adherens junctions (AJ) [3, 4], a complex founded on E-cadherin, the caretaker of epithelial integrity [5]. It is well established that loss of E-cadherin is sufficient to drive anchorage-independence in breast cancer cells in the context of oncogenic modulation of p53 [4] or activation of the PTEN/PI3K/AKT signaling axis [6, 7]. Because of cell-cell adhesion deficiency, ILC cells invade as single non-adhesive cells that - due to physical restriction - appear as collective invasive strands (reviewed in: [8]). ILC is mostly estrogen receptor (ER) positive with indolent growth characteristics, reflected by its low to intermediated grade. ILC has a tendency to present contra-lateral involvement and single cell dissemination patterns to sites such as the gastro-intestinal tract, ovaries, effusion fluids and leptomeninges [9]. Several studies have demonstrated that ILC cells display a specific, cell-intrinsic genomic profile and biochemistry driving their solitary survival [10]. Upon loss of AJ function and independent of ER or PI3K status, ILC cells show constitutive actomyosin contraction and AKT hyperactivation through growth factor receptor cues, which could explain their propensity for expansion in pleural and abdominal fluids (reviewed in: [11]). Although consensus is not complete, large and multi-parametric studies indicate that ILC patients have a worse survival after a prolonged (>15–20 years) follow-up when compared to non-ILC breast cancers [12, 13]. In sum, these traits indicate that metastatic ILC cells dampen their proliferation rate and promote cell survival pathways over extended time periods to facilitate diffuse cancer cell dissemination.

In non-cycling G0/G1 cells the retinoblastoma (Rb) tumor suppressor is hypo-phosphorylated and interacts with E2F factors to control repression of its target genes. Upon a mitotic stimulus or engagement of integrin-dependent anchorage, Rb is inactivated through cyclin-CDK-dependent phosphorylation, which disrupts its binding to the E2Fs (reviewed in: [14]). Rb-dependent cell cycle responses can be modulated by the inhibitor of DNA binding 2 (Id2), a helix-loop-helix (HLH) factor that acts as a regulator of E-protein function by binding and sequestering hypo- Rb through its pocket domains [15, 16]. In these studies, which were performed under anchorage-dependent two-dimensional conditions, upregulation of Id2 enhances cell proliferation and inhibits apoptosis. Nonetheless, it remained unclear how Id2-dependent control over cell cycle progression is instigated upon Id2 binding to hypo-phosphorylated Rb, and how this controls proliferation and anchorage independence of carcinoma cells.

Id2 expression has been linked to tumor initiation, growth, dissemination, and chemotherapy responses [17–20]. Increased Id2 levels in breast cancer correlate to poor prognosis [21], and a recent report has identified Id2 as a promoter of breast cancer colonization to the brain [22]. Also, Id2 expression has been implicated in the maintenance of breast cancer stemness and possible control over the transition from ductal carcinoma in situ (DCIS) to invasive cancer [23]. While Id2 controls tumor progression features, it is still unclear how its established ties to Rb-dependent cell cycle regulation affect tumor cell survival, anchorage independence, and metastasis. Importantly, the underlying mechanisms controlling Id2 expression during tumor progression are still elusive.

Here, we demonstrate that Id2 represents a key factor that promotes sustained cell cycle quiescence in human and mouse models of ILC. Our data provides a causal link from E-cadherin loss to a direct and p120/Kaiso-dependent transcriptional upregulation of Id2, which is exacerbated in the absence of cell-matrix engagement. Functionally, Id2 promotes a low proliferative indolent state in lobular breast cancer cells through inhibition of CDK4/6-Rb-dependent cell cycle progression, enabling anchorage independence and subsequent metastatic dissemination.

## Results

### E-cadherin deficient anoikis resistant breast cancer cells undergo a sustained G0/G1 arrest in anchorage-independent conditions

**Fig. 1:**
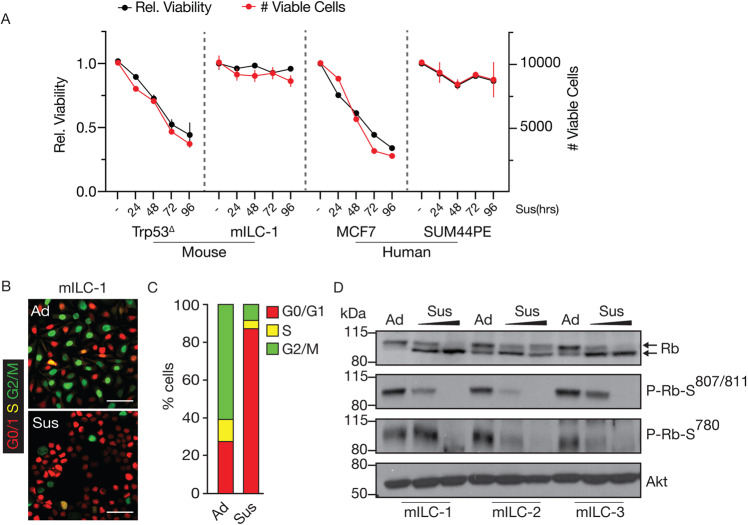
Anoikis resistant E-cadherin mutant breast cancer cells arrest in cell cycle phase G0/G1. **A** Relative viability (left-axis; %) and viable cell count (right-axis; #) in mouse and human E-cadherin positive (Trp53^∆^, MCF7) and E-cadherin negative cell lines (mILC-1, SUM44PE). Black trend lines show relative viability and red shows absolute viable cell numbers monitored in suspension. Note the decline in viability in the E-cadherin positive cell lines. Error bars show standard deviation. Error bars represent the standard deviation (SD). **B** Anchorage-independent mILC-1 cells are arrested in G0/G1. Shown are representative fluorescence images of adherent (Ad) and anchorage-independent (Sus/24 h) mILC-1 cells expressing the FUCCI-2 system. Scale bar = 50 μm. **C** mILC-1 cells in the G0/G1 (red), S (yellow), or G2/M (green) cell cycle phase (shown in **D**) were quantified (**E**). At least 500 cells were quantified per condition. Cells were cultured for 24 h in anchorage independent conditions before analysis. **D** Rb is dephosphorylated upon transfer to anchorage-independent conditions. Western blots showing the extent of Rb phosphorylation (Serine epitopes 780 and 807/811) in three different mILC cell lines. Upper arrow in the top blot indicates hyper-phosphorylated Rb; Lower arrow indicates hypo-phosphorylated Rb. Cells were cultured for either 12 or 24 h (Sus) in anchorage independent conditions. Akt was used as loading control. (All) Representative results are shown from at least three biological independent experiments.

ILC cells are resistant to anchorage-independent programmed cell death (anoikis), a hallmark that underlies their metastatic capacity in vivo (Fig. 1A; relative viability (left) and number of cells (right)) (reviewed in: [11]). For this study we initially used mouse ILC (mILC-1) cells, because they do not contain oncogene-addictive genetic aberrations in the RAS, RAF, BRAF or the PI3K/AKT or Rb pathways [24]. Under anchorage-independent conditions (Sus), metastatic mILC-1 cells cease to proliferate whilst retaining approximately 75% viability for several weeks [4, 25], suggesting that these anoikis resistant cells undergo a sustained G1/G0-arrested viable state. As expected, we observe that the majority (>90%) of viable cells arrest in G0/G1 when FUCCI-2 tagged cells are cultured in suspension conditions, whereas the adherent cells asynchronously proliferate, judged by the majority of mILC-1 cells in the S/G2/M phase (green or yellow cells; relative in %; Fig. 1B, C).

Because cell cycle arrest in G0/G1 is mediated through sequestering and inhibition of E2F transcription factors by hypo-phosphorylated Rb, we assessed the phosphorylation status of Rb under anchorage-independent conditions. In accordance with the observed anchorage-independent cell cycle arrest, Rb phosphorylation shifts from the hyper-phosphorylated (inactive) form under adherent conditions to the hypo-phosphorylated (active) in anchorage-independent suspended cells (Fig. 1D). Moreover, phosphorylation of the Rb Serine residues 780 and 807/811, which relies on CyclinD-CDK4/6 and CyclinE-CDK2 complexes, is reduced after 8 h and absent after 24 h of anchorage-independence (Fig. 1D). In short, anoikis-resistant mILC-1 cells transition towards a sustained G0/G1 cell cycle arrest under anchorage-independent settings.

### Id2 is upregulated during anchorage-independence to enable anoikis resistance in ILC

**Fig. 2:**
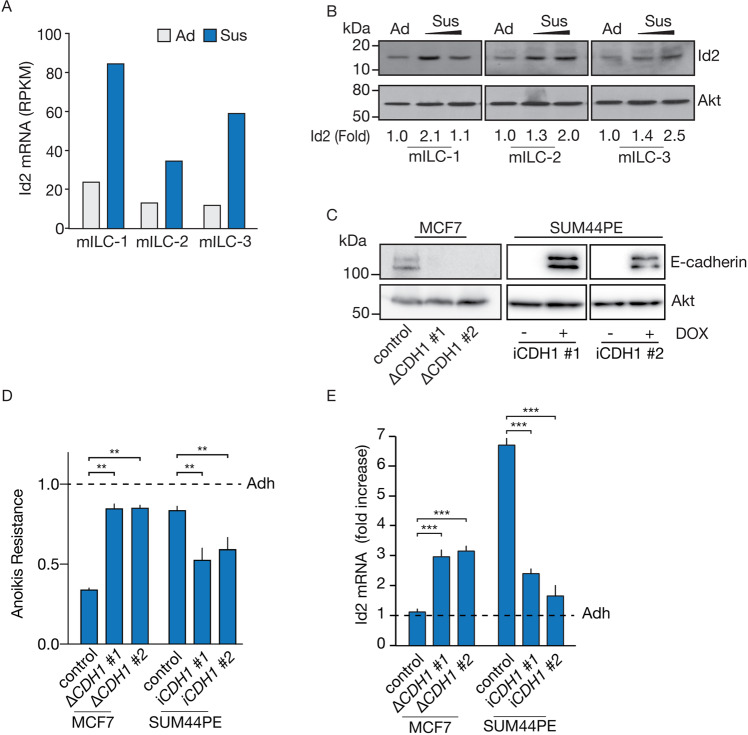
Id2 is transcriptionally upregulated in E-cadherin negative breast cancer cells in suspension. **A** Transcriptional upregulation of *Id2* in suspended mouse ILC (mILC) cells. *Id2* mRNA levels were quantified in three independent anchorage-independent mILC cell lines cultured in adherent (Adh) or suspension (Sus/24 h) conditions using mRNA sequencing. Expression is depicted as Reads per kilo base per million mapped (RPKM) values. **B** Western blot showing Id2 protein expression in cells from **A**. Akt was used as a loading control. Western blots were quantified by comparing pixel intensity of control compared to the condition. **C** Western blot analysis showing E-cadherin levels in isogenic MCF7 control (WT) *versus* two MCF7 knock-out clones (MCF7::Δ*CDH1)* (left panel), and two SUM44PE ILC cells transduced with a DOX-inducible full length and wild type E-cadherin (SUM44PE::i*CDH1*)(right panel). Akt was used as a loading control. **D** Anoikis resistance assay performed in cell lines from **C**. MCF7 control (WT) and MCF7::Δ*CDH1* isogenic clones 1 and 2 were kept in anchorage independent conditions for 96 h before harvesting, whereas SUM44PE were kept in anchorage independent conditions for 168 h. Note the approx. 2-fold increase in both MCF7::Δ*CDH1* clones *versus* MCF7 control, and approx. 1.5-fold reduction in anoikis resistance for SUM44PE::i*CDH1* cell line and 2. The Student’s *t* test was used to determine statistical significance. ***p < 0.001, **p < 0.01. Error bars show the standard deviation (SD). **E** E-cadherin loss induces *Id2* expression in suspended cells *versus* adherent cells. Shown are the RT-qPCR mRNA expression fold increases for *Id2* in isogenic MCF7 control and two E-cadherin knock-out (∆*CDH1*) cell lines and the ILC cell line SUM44PE:i*CDH1*. Note the approximate 3 to 5-fold induction of *Id2* mRNA expression in the E-cadherin mutant cells in anchorage-independent conditions (Sus). Also note that E-cadherin reconstitution in SUM44PE::i*CDH1* upon DOX administration reduces the *Id2* mRNA expression in control SUM44PE::i*CDH1* cells by an average approximate of 70%. Relative *Id2* mRNA expression levels were normalized to either GAPDH or ACTB. MCF7 control (WT), MCF7:: Δ*CDH1* were kept in anchorage independent conditions for 96 h, and SUM44PE for 168 h/7 days, before harvesting and analysis. The Student’s *t* test was used to determine statistical significance. ****p < 0.0001, ***p < 0.001, **p < 0.01. Error bars show the standard deviation (SD). (All) Representative results are shown from at least three independent experiments.

To identify a mechanism that unites concomitant regulation of cell cycle arrest and anoikis resistance driven by E-cadherin loss, we isolated mRNA from three anoikis resistant and ER^NEG^ mILC cell lines cultured under anchorage-independent conditions and performed genome-wide mRNA sequencing as described previously [24]. In conjunction with our previous results [26], we detected that the helix-loop-helix (HLH) factor *Id2* is upregulated in suspended mILC cells (Fig. 2A). Id2 was prioritized because it is an established regulator of cancer proliferation and Rb-dependent cell cycle progression [15, 16]. Western blot analysis of mILC cell lines confirms an approximate 2 to 3-fold increase in Id2 protein levels under anchorage-independent conditions, which increases over time (Fig. 2B).

Next, we analyzed if E-cadherin is causal to the regulation of *Id2* expression in ER positive human breast cancer cells. For this we compared the E-cadherin expressing MCF7 wild type (WT) cell line *versus* two isogenic MCF7 E-cadherin knockout (∆*CDH1*) clones (Fig. 2C; left panel). Furthermore, we reconstituted E-cadherin expression using a Doxycyclin (DOX)-inducible system (i*CDH1)* in SUM44PE, an established ILC cell line, and generated two SUM44PE::i*CDH1* cell lines. (Fig. 2C; right panel and Supplementary Fig. 1). As expected, we observe that E-cadherin controls anoikis resistance. Inducible E-cadherin protein expression in SUM44PE::i*CDH1* cells results in a significant reduction in anoikis resistance for both inducible clones, whereas E-cadherin loss in MCF7 induces anoikis resistance in both isogenic *CDH1* cell lines (Fig. 2D). Importantly, anoikis resistance upon E-cadherin loss correlates to increased *Id2* mRNA levels. MCF7::Δ*CDH1* cells reveal an average approximate 3-fold increase of *Id2* mRNA in suspension (Fig. 2E), whereas inducible restoration of E-cadherin expression in SUM44PE cells results in an average 3-fold downregulation of *Id2* expression in suspension (Fig. 2E).

In short, our data demonstrate that E-cadherin loss causes Id2 upregulation in breast cancer cells under anchorage independent conditions.

**Fig. 3:**
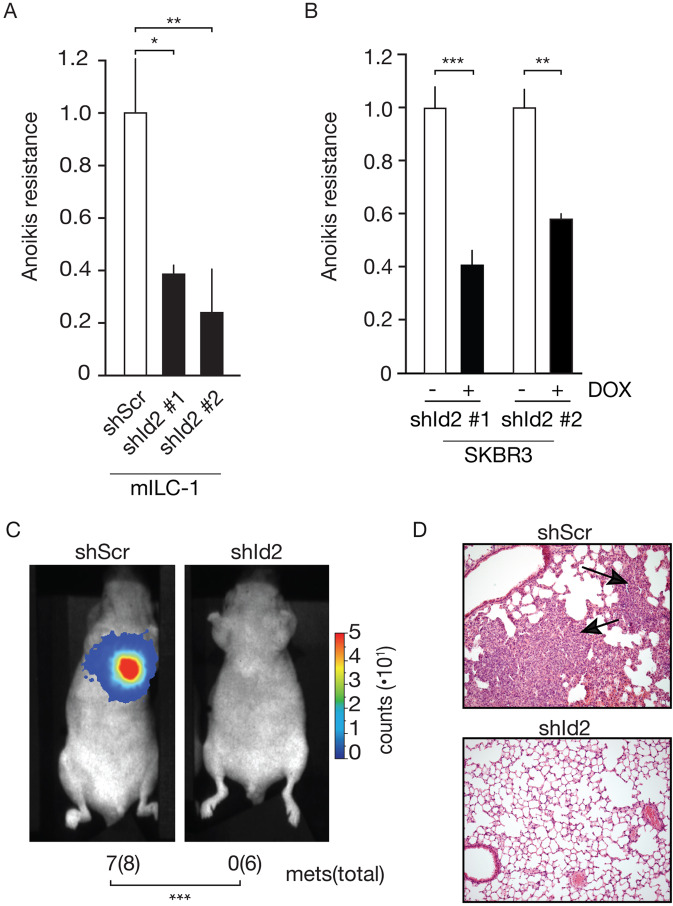
Id2 promotes anoikis resistance and metastatic colonization in E-cadherin deficient breast cancer cells. Id2 controls anchorage-independent survival. Shown are the levels of anoikis resistance upon knockdown of Id2 in mouse mILC (**A**) or E-cadherin mutant human SKBR3 (**B**) after 24 or 96 h in suspension respectively. Scrambled shRNA (shScr) and Doxycycline (DOX) controls are depicted in white bars. The Student’s *t* test was used to determine statistical significance. ***p < 0.001, **p < 0.01, *p < 0.05. Results are expressed as mean and error bars represent standard deviation (SD). All experiments were repeated at least 3 times. **C**, **D** Id2 expression is necessary for lung colonization. Shown are bioluminescence signals (relative counts) from luciferase-tagged mILC cells injected intra-venously (iv) in the tail vain of recipient Nude mice (**D**). The effect of Id2 knockdown (shId2) was assessed and compared to controls (shScr). The number of mice showing lung colonization 4 weeks post injection are depicted below, together with the total number of mice injected in parenthesis. A two-tailed Fisher’s exact test was used to determine statistical significance. ***p < 0.001. Panel **D** shows a representative H&E stained lung sections (magnification 100X) of the mice depicted in **C**. Arrows point to metastatic outgrowth. For all mouse experiments, animals were randomized, and observers blinded for the experimental conditions.

To investigate if Id2 contributes to anchorage-independent survival (anoikis resistance), we performed Id2 knock-downs in mILC using two independent shRNAs (Supplementary Fig. 2A), which results in a 2 to 5-fold decrease in anoikis resistance (Fig. 3A, *p* < 0.001). Apoptosis was verified by blotting for cleaved Caspase-3 (Supplementary Fig. 2B). Comparable to the mILC cells, we show that Id2 knockdown in E-cadherin mutant human SKBR3 cells using two independent Doxycycline (DOX)-inducible shRNA sequences (Supplementary Fig. 2C) results in an approximate 50–60% decrease in anoikis resistance (Fig. 3B).

To probe the in vivo consequences of Id2-dependent anchorage-independence, we performed experimental metastasis assays. We opted for intravenous injections to effectively assess lung colonization capacity as a surrogate readout for the metastatic potential of disseminated cells, because we aimed to uncouple dependency of distant colonization from primary tumor growth. These in vivo assays demonstrate that Id2 knockdown fully prevents lung colonization compared to controls (0/6 versus 7/8 mice, *p* < 0.0001; Fig. 3C, D), indicating that Id2 is required for metastatic colonization of E-cadherin deficient breast cancer cells.

Next, we analyzed if expression of Id2 in E-cadherin positive cells is sufficient to induce anoikis resistance. For this, we overexpressed mouse Id2 in MCF7 wild type cells and quantified apoptosis in suspension cultures using FACS. Interestingly, Id2 overexpression induced a modest but significant 15% reduction of cells undergoing anoikis after 96 h. which was accompanied by a reduction in RB^S807/811^ phosphorylation levels (Supplementary Fig. 3A, B).

Our data thus show that Id2 is essential for anoikis resistance in E-cadherin mutant ILC cells, and suggest that Id2 is sufficient sustain partial anoikis resistance in E-cadherin expressing cells.

### Id2 is a p120-responsive Kaiso target gene in E-cadherin deficient breast cancer cells

**Fig. 4:**
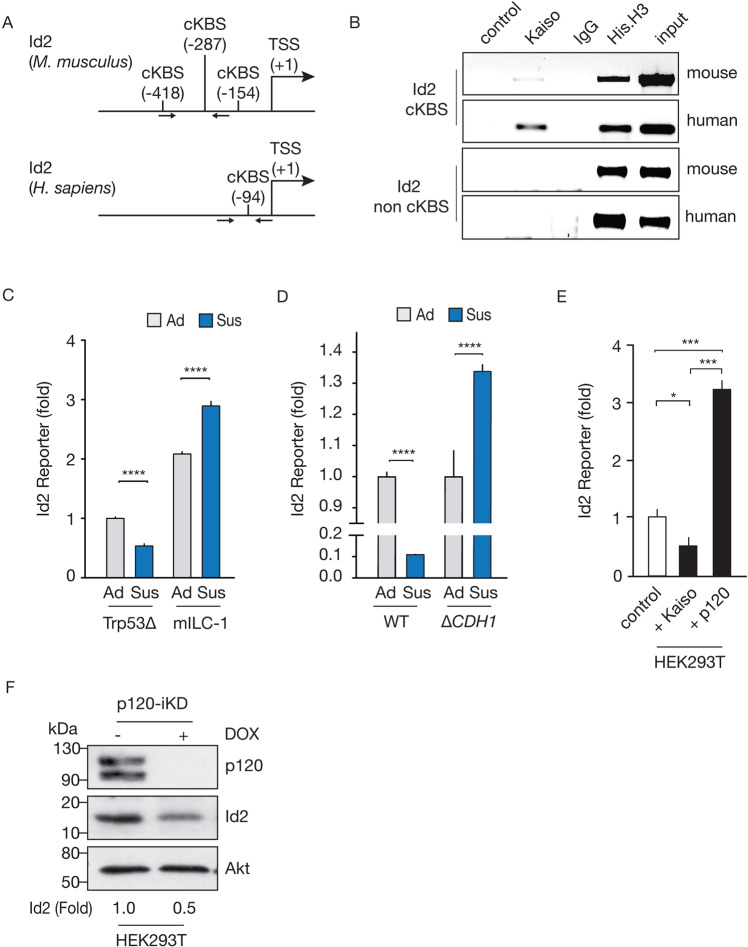
Id2 is a p120-responsive Kaiso target gene. **A** Schematic overview of the promoter regions of mouse and human *Id2* depicting the position of the canonical cKBS *(CTGCNA)* relative to the transcription start site (TSS). *Id2*-specific primers used in (B) are indicated by arrows. **B** *Id2* is a Kaiso target gene. Chromatin immunoprecipitated (ChIP) samples from mILC or human E-cadherin deficient MDA-MB-231 cells were amplified using an *Id2*-specific PCR using primary flanking the cKBS consensus sites (top panels). Negative controls consisted of *Id2*-specific non-cKBS primers (bottom panels), no input/Kaiso antibodies (control), IgG mouse antibodies (IgG) and a positive control was based on an anti-H3 antibody ChIP. **C**, **D** E-cadherin mutant cells upregulate Id2 under anchorage-independent conditions. Mouse syngeneic breast cancer and isogenic MCF7 control (WT) *versus* MCF7::Δ*CDH1* were transduced with an *Id2*-mCherry reporter system and fluorescence was quantified using FACS analysis in adherent (Ad, gray bars) and suspension (Sus, blue bars). Timing of suspension conditions: mouse cells 24 h and human cells 96 h. Note the increase in mCherry fluorescence in the E-cadherin mutant mouse (mILC-1) and human MCF7::Δ*CDH1* cells compared to the E-cadherin expressing cells (Trp53^∆^ and MCF7 WT) (D). ****p < 0.0001. **E**, **F** *Id2* is a Kaiso target gene regulated by p120. Quantification of *Id2* reporter activity based on transcriptional luciferase activity. HEK293T cells were co-transfected with either an empty pCDNA vector (control), Kaiso, or p120-1A and cultured in adherent conditions. **E** Note the 2-fold decrease in Kaiso co-transfected cells and the 3-fold increase in reporter activity in cells co-transfected with p120. The western blot depicts the effect of p120 depletion using Doxycycline (DOX)-inducible knockdown (iKD) of both p120 isoforms on Id2 protein expression. Note the approximate 3-fold reduction in Id2 protein expression upon DOX addition. For **F** Western blots were quantified by comparing the pixel intensity of controls *versus* experimental conditions. (**All**) Representative experiments are shown from at least three independent biological replicates. For **C**–**E**, the mean and standard deviation are shown. The Student’s *t* test was used to determine statistical significance. ***p < 0.001, **p < 0.01, *p < 0.05.

Our previous studies had identified *Id2* as a candidate Kaiso target gene due to the presence of canonical Kaiso binding sites (cKBS; *TCCTGCNA*) in mouse *Id2* [26]. Further analysis confirmed the presence of a cKBS in human promotor sequences (−500bp to +100 bp) (Fig. 4A). Chromatin immunoprecipitation (ChIP) assays using monoclonal Kaiso antibodies combined with an *Id2*-specific PCR confirms that *Id2* is a Kaiso target gene in mouse and human E-cadherin deficient breast cancer cells (mILC-1 and MDA-MB231) (Fig. 4B). We next cloned approximately 1 kb of the endogenous mouse *Id2* promoter (Id2-pro1036) [27] upstream of a mCherry expression cassette to test promotor activity under anchorage-independent conditions. Using mouse syngeneic (Trp53^∆^ *versus* mILC-1) and isogenic human MCF7 cells (WT *versus* ∆*CDH1*) models, we observe that loss of E-cadherin increases *Id2* reporter activity when transferring cells to anchorage independent conditions (Sus; Fig. 4C, D). Using a luciferase-based Id2 reporter construct transfected into HEK293T cells, we show that transcriptional *Id2* responses are reduced 2-fold by exogenous Kaiso expression (Fig. 4E). We also observe an approximate 3-fold increase in *Id2*-specific reporter activity upon co-expression of p120-1A (Fig. 4E), indicating that *Id2* transcription is de-repressed by nuclear p120/Kaiso complex. Moreover, DOX-inducible p120-specific knockdown (p120-iKD) leads to an approximate 3-fold decrease in Id2 protein levels (Fig. 4F). Together, these results establish Id2 as a novel and p120-responsive transcriptional Kaiso target on its canonical target site and provide a mechanistic rationale for the Id2-dependent pro-metastatic control over anoikis resistance in E-cadherin mutant breast cancer cells.

### Id2 inhibits Rb-dependent cell cycle progression in the absence of anchorage

Because Id2 can modulate cell cycle responses through the Rb pocket proteins [15, 16], we reasoned that upregulation of Id2 promotes viability through a sustained G0/G1 arrest in anchorage independent ILC cells. We therefore performed co-immunoprecipitation studies on lysates from suspended anoikis resistant cells to confirm that Id2 binds hypo-phosphorylated Rb. In line with published data [15], we could effectively pull-down hypo-phosphorylated Rb with Id2 antibodies (Supplementary Fig. 4A). Unexpectedly, we find that Id2 is mainly localized to the cytoplasm of mILC-1 cells in both adherent and anchorage independent conditions (Supplementary Fig. 4B–E). Therefore, our data suggest that under these conditions, the Id2-Rb interaction prevents phosphorylation of Rb by cyclin-dependent kinases, thereby attenuating cell cycle responses of E-cadherin mutant ILC cells.

**Fig. 5:**
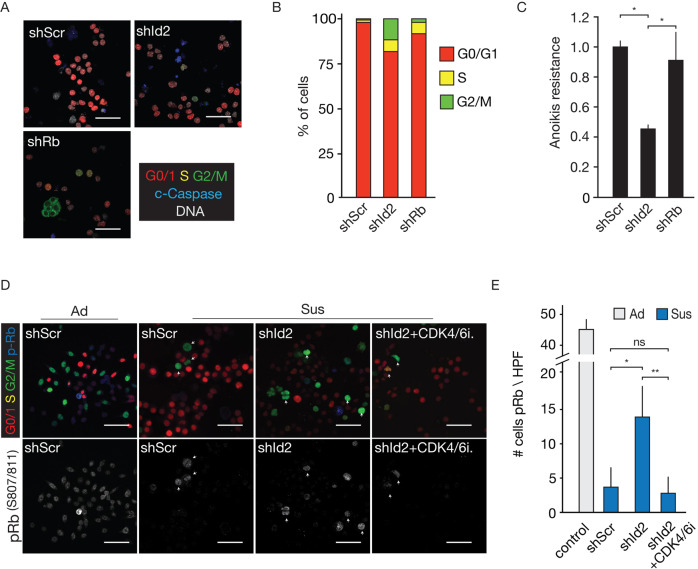
Id2 controls inhibition of cell cycle progression. **A**–**C** Id2 controls viability through inhibition of cell cycle progression under anchorage-independent culture conditions. FUCCI-2-tagged mILC-1 cells were cultured in suspension for 24 h and stained for apoptotic cells using cleaved Caspase-3 (blue; **A**). Cell cycle stages of mILC-1 cells in which either Id2 or Rb expression were inhibited, were quantified in **B** and viability was assessed in parallel using FACS analysis for Annexin-V and propidium iodine incorporation **C**. **C** The Student’s *t* test was used to determine statistical significance. **p < 0.01, *p < 0.05. Error bars depict the standard deviation. Size bar = 5 µm. **D**, **E** Id2 depletion causes an increase in cycling cells in a Rb and CDK4/6-dependent manner. Phosphorylated Rb (pRb Ser807/811) IF staining of Adh and Sus (24 h) mILC-1 cells (**D**). Note the absence of p-Rb signals (blue signals) and the reduction of G2/M cells in the Id2-depleted condition in the presence of the CDK4/6 inhibitor ribociclib (CDK4/6i; 3 µM). The number of cells expressing p-Rb from **D** are quantified in **E**. Outlines indicate cells with high pRb staining in G2/M phase. **E** HPF High Power Fields, showing one microscope image in high resolution. Five HPFs containing at least 120 cells per cell cycle state were quantified. **E** The Student’s *t* test with Welch’s correction was used to determine statistical significance. **p < 0.01, *p < 0.05. Error bars depict the standard deviation. (All) Representative experiments are shown from at least three biological independent replicates.

We next confirmed the impact of Id2 or Rb knockdown (Supplementary Fig. 5A) on adherent growth using 2D colony formation assays. In full agreement with published results, we observe that Id2 inhibition in mILC cells results in a marked decrease of proliferation under conventional 2D culture conditions (Supplementary Fig. 5B, C). As expected, loss of Rb has no impact on colony formation, as Rb is already in its inactive hyper-phosphorylated form in most adherent cells. Next, we probed if Id2 knockdown (shRNA) influenced cell cycle progression under anchorage-independent culture conditions. Since Id2 knockdown causes a pronounced induction of anoikis in E-cadherin mutant cells (Fig. 3), we were surprised to observe an increase in cycling and apoptotic cells when cultured in suspension (Fig. 5A, B). Knockdown of Id2 results in an approximate 17% expansion of cells in G2/M and S phase that coincides with an apparent paradoxical decrease in the percentage of viable cells (Fig. 5B, C). In contrast, shRNA depletion of Rb induces a modest increase in cycling cells (Fig. 5A, B), but with no significant effect on anchorage-independent viability (Fig. 5C).

Following this, we assessed whether the induction of cell cycle progression in the absence of Id2 was CDK4/6 dependent. Under anchorage-independent conditions, Id2 knockdown cells show an approximate 5-fold increase in Ser 807/811 Rb phosphorylation, an effect that can be largely abolished by culturing cells in the presence of a CDK4/6 inhibitor (Fig. 5D, E). Because the increase in G2/M cells and the reduction of viability coincides with an approximate 3-fold cumulative increase of phospho-γH2AX positive DNA damage foci in Id2-depleted cells in G2/M (Supplementary Fig. 6A, B), we conclude that upregulation of Id2 prevents a replication stress-induced accumulation of DNA damage in suspended cells.

Together, these data show that in E-cadherin mutant lobular breast cancer cells, Id2 binds to hypo-phosphorylated (active) Rb to dampen cell cycle progression. Further upregulation of Id2 under anchorage-independent conditions blocks cell cycle progression, which prevents accumulation of DNA damage and fosters anoikis resistance.

### Id2 expression is increased in E-cadherin mutant primary human organoid ILC models

**Fig. 6:**
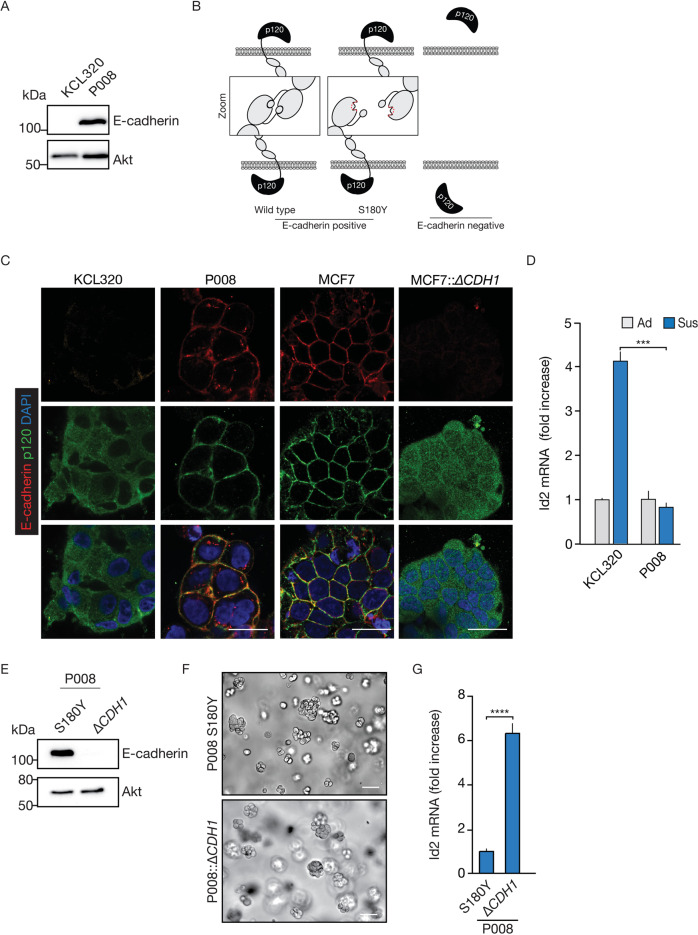
Id2 is upregulated in PDO-PDX human ILC models. **A** Western blot analysis showing E-cadherin levels in the classical ILC KCL320 and in the E-cadherin expressing non-classical ILC PDO model P008 (top panel). Akt was used as loading control (bottom panel). **B** Schematic representation of the *CDH1* mutation S180Y and the consequences for cell-cell contacts. Note that although E-cadherin S180Y is unable to establish homotypic in trans interactions with adjacent cells, it retains p120-catenin at the cell membrane. **C** Immunofluorescence showing expression and localization of E-cadherin (red; top panels), p120-catenin (p120; green, middle panels). Merged images are shown in the bottom panels. Note the cytosolic p120 expression in E-cadherin deficient cells *versus* the co-localization of E-cadherin and p120 in E-cadherin-expressing P008 ILC cells. Isogenic MCF7 and MCF::Δ*CDH1* cell lines were used as a controls. Size bar = 5 µm. **D** RT-qPCR analysis of *Id2* mRNA upregulation in anchorage-independent cells (Sus/168 h). Note the 4-fold upregulation of *Id2* in KCL320 suspended cells compared to adherent conditions (Ad). Relative *Id2* mRNA expression levels were normalized to either GAPDH or ACTB. The Student’s *t* test was used to determine statistical significance. ****p < 0.0001, ***p < 0.001. Error bars show the standard deviation. **E** Western blot analysis showing E-cadherin levels in the isogenic P008 and P008::Δ*CDH1* PDO models (top panel). Akt was used as a loading control (bottom panel). **F** Representative differential interference contrast microscopy images of P008 and P008::Δ*CDH1* cultured in 3D basement membrane extract-derived matrix. Note the noncoherent “grape-like” phenotypic appearance of both PDO models. Size bar = 50 µm. **G** RT-qPCR analysis of *Id2* mRNA upregulation in anchorage-independent cells. Note the approximate 6-fold upregulation in P008::Δ*CDH1* cells compared to P008 suspended cells. Both organotypic cell lines were kept in suspension for 7 days before harvesting and analysis. Relative *Id2* mRNA expression levels were normalized to either GAPDH or ACTB. The Student’s *t* test was used to determine statistical significance. ****p < 0.0001, ***p < 0.001. Error bars show the standard deviation. (All) Representative results are shown from at least three independent experiments.

We next employed two primary human metastatic patient-derived organoid (PDO) ILC models to assess anchorage independent Id2 expression in 3D. We compared KCL320, a classical E-cadherin negative ILC PDO model to P008, an E-cadherin positive ILC PDO model that contains a S180Y mutation in *CDH1* (Fig. 6A, B). The extracellular *CDH1* mutation in P008 is predicted to render a full-length E-cadherin molecule that cannot undergo homotypic interactions in trans, thus leading to loss of cell-cell adhesion (Supplementary Fig. 7). Using immunofluorescence, we confirm that p120-catenin is mostly localized to the cytosol in KCL320, while P008 displays co-localization of E-cadherin and p120-catenin at the plasma membrane (Fig. 6C). E-cadherin localization in P008 is fragmented when compared to E-cadherin in MCF7 (Fig. 6C), a likely consequence of the inability to engage in homotypic interactions that may result in lateral clustering [28]. In line with our findings in breast cancer cell lines (Fig. 2), we observe a 4-fold increase of *Id2* mRNA levels in the E-cadherin negative KCL320 upon transfer to anchorage-independence, but not in the E-cadherin expressing ILC PDO P008 (Fig. 6D). We next used CRISPR/Cas9 to knock-out mutant E-cadherin expression from P008 (Fig. 6E) and observe that loss of E-cadherin expression in P008::Δ*CDH1* cells does not impact the typical non-coherent ILC-type 3D structures (Fig. 6F). However, transfer of the isogenic P008 and P008::Δ*CDH1* PDO models to anchorage independence showed that E-cadherin loss induces a 5-fold increase of *Id2* mRNA levels (Fig. 6G). In short, anchorage independent *Id2* mRNA upregulation is causally linked to E-cadherin expression in PDO breast cancer models.

### Id2 localizes cytoplasmic in invasive lobular breast carcinomas

**Fig. 7:**
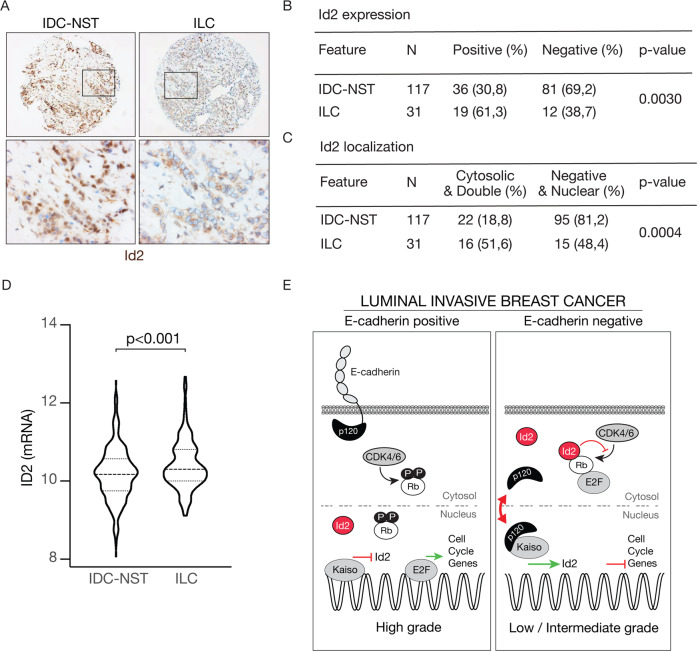
Increased cytosolic localization of Id2 characterizes ILC. **A**–**C** Cytosolic Id2 expression is prevalent in ILC. Shown are representative tissue micro array (TMA) cores (top panels) from IDC-NST and ILC stained for Id2 expression (**A**) (brown). A total of 148 invasive breast cancer samples were scored as binary output and quantified in **B**. Note the overall increase in Id2 expression when comparing ILC versus IDC-NST in **B**, and the increase in the number of cases that express cytosolic Id2 when comparing ILC versus IDC-NST (**C**). Pearson Chi-square tests were used to determine statistical significance. *P*-values are stated in the table. **D** Shown are Id2 mRNA expression levels in the TCGA dataset. Wilcoxon rank tests were used to assess statistical significance. In total 533 breast cancer samples were analyzed (388 IDC-NST *versus* 145 ILC). **E** Id2 controls sustained indolent proliferation in ILC. Cartoon depicting a proposed model for E-cadherin dependent regulation of cell cycle progression. Show are E-cadherin expressing (left panel) and E-cadherin negative breast cancer (right panel). Note the consequences of E-cadherin loss (right panel), inducing cytosolic and nuclear translocation of p120 and a subsequent de-repression of Id2 expression by Kaiso. Id2 will accumulate in the cytosol, where it binds hypo-phosphorylated Rb and inhibits phosphorylation of Rb by CDK4/6. Cell cycle progression will be dampened, underpinning the indolent and low-grade features of E-cadherin mutant ILC. Under anchorage independent conditions (dissemination), Id2 expression is further increased due to a nuclear influx of p120 [26], leading to a full prevention of Rb phosphorylation, clustering E2F, and inhibition of cell cycle progression in G0/G1.

We next analyzed a cohort of 148 invasive breast cancer samples (Supplementary Table 1) and compared Id2 expression between IDC of no specific subtype (IDC-NST) and ILC using immunohistochemistry (IHC). In line with our data from the primary and cell line ILC models, we observe that ILC has a higher percentage of cases that express Id2 when compared to IDC-NST (Fig. 7A, B; *p* = 0.003). Analysis of the publicly available TCGA database reveals that *Id2* mRNA expression is indeed elevated in ILC when compared to IDC-NST (ER^POS^:HER2^NEG^ samples; *p* < 0.001) (Fig. 7D). As expected, we did not find indications that Id2 expression is prognostic between IDC-NST and ILC when analyzing several publicly available breast cancer datasets (TCGA, METABRIC, Michaut [29] and Metzger [30]) (Supplementary Fig. 8). Using a clinical breast cancer tissue microarray (TMA), we analyzed subcellular distribution of Id2 in 148 invasive breast cancers and observe that ILC cases more often display cytosolic Id2 localization compared to IDC-NST (Fig. 7C, *p* = 0.0004). These clinical findings concur with our in vitro data that Id2 predominantly localizes in the cytoplasm of ILC cells.

In conclusion, loss of E-cadherin induces a p120/Kaiso-dependent upregulation of Id2, a factor that is essential for anchorage independence and binds hypo-phosphorylated Rb in the cytosol, leading to CDK4/6-dependent cell cycle repression in ILC (Fig. 7E).

## Discussion

It is well-established that luminal ER expressing breast cancers tend to relapse with a longer latency than non-luminal tumors [31]. Interestingly, despite the absence of lymph node involvement and presenting smaller low to intermediate grade tumors, patients with luminal breast cancers will develop distant recurrences [32]. Since large human and mouse studies have suggested that breast cancer dissemination is an early event [33, 34], these combined findings strongly indicate that low proliferative and anoikis-resistant cancer cells are at the basis of non-lymphatic dissemination and long-latency relapses. In this study, we employed both human and mouse ILC models as a paradigm to study the mechanism that drives survival of breast cancer cells during periods of sustained proliferative quiescence, because it might be essential for dormancy and long-term relapses of indolent luminal breast cancer. ILC is renowned for harboring all the hallmarks of an indolent systemic disease. First, low grade ILC invades as single non-cohesive cells that are intrinsically anoikis resistant (reviewed in: [35]). Second, an ILC diagnosis predicts micro-metastasis in breast cancer [36]. Third, ILC tends to present itself at contralateral sites [37], and examples of metastatic presentation without a primary tumor have been reported [38, 39]. In line with these disease characteristics, our study demonstrates that indolent ILC cells dampen their proliferative responses upon E-cadherin loss through direct transcriptional de-repression of Id2-dependent cell cycle inhibition, thereby sustaining cell viability in suspension. Given that we demonstrate causality in ER^POS^ and ER^NEG^ models of human and mouse origin, it also indicates that this mechanism operates independent of estrogen receptor function.

High Id2 levels are associated with terminally differentiated mammary cells, coinciding with β-casein expression in the mammary gland at day 12 of pregnancy [40]. In line with this, it was reported that during mammary gland development, nuclear retention of Id2 is essential for homeostatic differentiation [41]. As such, it appears that Id2 is important for inhibition of differentiation and simultaneous activation of proliferation in the context of normal organ development and function. Seminal studies have shown that Id2 can positively control proliferation of many cancer cell types in 2D, *i.e*. anchorage-dependent conditions [16, 42]. In adherent dividing cells, the vast majority of Rb is in the hyper-phosphorylated state, allowing E2F transcription factors to drive cell cycle progression [43]. The Rb-dependent proliferative functions of Id2 were identified in U2OS cells, where exogenous Id2 overexpression could overcome serum dependency for growth. The group that first reported these findings moreover demonstrated that Id2 depended on its helix-loop-helix domain for binding the E1A/Large T pocket of hypo-phosphorylated Rb [44], and concluded that this led to an increase in hyper-phosphorylated Rb, driving cell cycle progression [15]. However, because several viral oncoproteins also bind Rb in this pocket, we consider it is more likely that binding of hypo-phosphorylated Rb by cytosolic Id2 in suspended non-proliferative ILC cells hinders initial CDK4/6-dependent phosphorylation of Rb and subsequent progression beyond the G1 restriction point. Conversely, because overexpression of Id2 in attached U2OS cells leads to a seemingly paradoxical observation of increased proliferation that coincides with decreased levels of Cyclin D1 and Cyclin D1/CDK complexes [16], it is also possible that cell cycle progression in attached cells, and in the presence of high Id2, may be partly independent of Rb binding, or solely dependent on p16^INK4A^ activity [45].

Our studies demonstrate that loss of E-cadherin leads to a p120/Kaiso-dependent relief of *Id2* transcriptional repression, a phenomenon that is enhanced in the absence of anchorage. Although our data fully support the reported binding of Id2 to hypo-phosphorylated Rb, we hypothesize that in anchorage-independent carcinoma cells, formation of the Id2-Rb complex prevents CDK4/6-dependent Rb phosphorylation by retaining or accumulating Rb in its hypo-phosphorylated active form. Supporting this is the fact that loss of Id2 leads to an increase in Rb phosphorylation and the number of cycling cells in ILC cells in suspension cultures. Second, low to intermediate grade ILC is strongly associated with overexpression of Cyclin D1 [46–48], a regulatory subunit of G1/S phase transition and entry into the S-phase by the cyclin-dependent kinases CDK4/6. This feature has been a long-standing and enigmatic paradox in breast cancer; how can high expression levels of Cyclin D1 in ILC be united with a low to intermediate grade breast cancer type? In light of this question and our data, we postulate that cytosolic Id2 functions as a CDK4/6 antagonist through binding to hypo-phosphorylated Rb and subsequent dampening of cell cycle progression in ILC cells. In this context, Id2 therefore might function similarly to the small molecule CDK4/6 inhibitors, inducing compensatory upregulation of Cyclin D1. Supporting this are observation that high Cyclin D1 expression levels correlate with long-term prognosis in ILC [49], and the finding that the CDK4/6 inhibitor abemaciclib induces upregulation of Cyclin D1 in luminal-type ER+ and lobular-type breast cancer cell lines [50]. Also, our findings in the mILC models and other comparable drug response studies using palbociclib [51], point to the notion that enhanced responses by luminal breast cancer cells are independent of HER2 status and ER function. Overall, we presume that an increase in Id2 levels during ILC anchorage-independence will halt cell cycle progression at G0/G1 and as such promote invasion and dissemination. Subsequent long-term quiescence or dormancy at specific metastatic niches could then be fostered through distinct and as yet unidentified ECM cues that propel high levels of Id2.

In closing, our study provides novel mechanistic insight and a conceptual rationale for the control over indolent proliferation of ILC, cellular quiescence and cycle re-entry upon cell anchorage. We propose that inactivation of E-cadherin, a lobular histology, and cytosolic Id2, should be considered as criteria to include ER^NEG^ and HER2^POS^ ILC patients for clinical CDK4/6 breast cancer intervention trials.

## Material and methods

### Cell line and organoid cultures

Mouse cell lines Trp53^∆^-3 and mILC1 (Cdh1^∆^;Trp53^∆^) have been generated from mammary tumors that developed in *K14cre;Cdh1*^*F/F*^*;Trp53*^*F/F*^ female mice and cultured as described previously [4]. Similarly, mouse cell lines mILC2 and mILC3 were generated from mammary tumors that developed in *Wcre;Cdh1*^*F/F*^*;Trp53*^*F/F*^ *female mice* [25]. 293T, MCF7, SKBR3, MDA-MB 231 and SUM44PE cell lines were obtained from the American Type Culture Collection (ATCC), STR type verified by PCR and cultured as described previously [52]. Generation of E-cadherin knock-out MCF7::∆*CDH1* and Trp53^∆^::∆*Cdh1* cells using CRISPR-Cas9 editing has been described previously [53]. Generation of additional cell lines is described in the supplementary methods. Derivation, establishment, and culturing of PDO models KCL320, P008 and P008::Δ*CDH1* is described in detail in the supplementary methods.

### Antibodies and reagents

The following primary antibodies were used for western blot analysis: monoclonal rabbit Id2 D39E8 (1:1,000; #3431; Cell Signaling Technology), polyclonal rabbit Id2 C-20 (1:1,000; sc-489; Santa Cruz Biotechnology), polyclonal rabbit Rb M-153 (1:1,000; sc-7905; Santa Cruz Biotechnology), rabbit monoclonal phospho-Rb Ser 780 (1:2000; #9307; Cell Signaling Technology), monoclonal rabbit phospho-Rb Ser 807/811 (1:2,000; #9308; Cell Signaling Technology), monoclonal mouse E-cadherin (ECH6; 1:1000; #MSK033; Zytomed); purified mouse p120-Catenin (Clone98; 1;1000; #610134; BD Biosciences), polyclonal rabbit GFP (1:1000; sc-8334; Santa Cruz Biotechnology), and polyclonal goat Akt1 C-20 (1:1,000; sc-1618; Santa Cruz Biotechnology). The following primary antibodies were used for Immunofluorescence (IF): monoclonal rabbit phospho-Rb Ser 807/811 (1:100; #9308; Cell Signaling Technology), polyclonal rabbit Cleaved Caspase-3 (Asp175) (1:50; #9661; Cell Signaling Technology.), monoclonal TRITC-conjugated mouse E-cadherin (clone 36, 1:50; BD560064; BD Biosciences), monoclonal mouse phospho-Histone H2A.X (Ser139) (clone JBW301, 1:1,000; #05-636; EMD Millipore), monoclonal mouse E-cadherin (ECH6; 1:200; #MSK033; Zytomed); purified mouse p120-Catenin (Clone98; 1;200; #610134; BD Biosciences). immunohistochemistry (IHC) antibodies: Monoclonal mouse Id2 (clone OTI10C3; 1:25,000; TA500183; Origene Technologies), Monoclonal rabbit phospho-Rb Ser 807/811 (1:100; #9308; Cell Signaling Technology).

### Plasmids

Lentiviral and inducible p120-specific shRNA constructs (p120-iKD) have been described previously [52]. Sequences were verified by Sanger DNA sequencing. Additional cloning methods and primer sequences are described in detail in the supplementary methods.

### Id2 reporter assay

HEK 293T cells were cultured and transfected with 100 ng pGL4-Id2pro-1036 and 5 ng pRL-Renilla in combination with either 400 ng empty vector (pcDNA3.1) or pC2-eGFP-p120-1A or pcDNA3.1-Kaiso using XtremeGene9 (Roche) Reporter activity was measured using the Dual-Luciferase Reporter kit (Promega) on a Lumat LB9507 Luminometer (Berthold Technologies) according to manufacturer’s instructions. Cell lines transduced with the Id2-mCherry reporter viruses were cultured in anchorage-independent conditions for the indicated time periods and fluorescence was quantified by flowcytometry using a FACS Celesta (BD Biosciences). Data was processed using the FACSdiva software (Version 8.0) and analyzed in Prism (Version 8.0).

### Kaiso Chromatin Immunoprecipitations (ChIP) and PCR

ChIP procedures and PCR were done as previously described [54]. The following primers were used for amplification of the target promoter sequences: mouse Id2: FW; 5′-CAA ATG TGA CTT CCC AAA AGC-3′ and REV; 5′-GCC TTA TTC CAA TTC CCA GA-3′. Human Id2: FW; 5′-AGC CCC GCA CTT ACT GTA CT-3′ and REV; 5′-CTT CCC TTC GTC CCC ATT-3′. The following primer sequences were used as negative control: mouse Id2: FW; 5′ – AGG ATC CCG GTG CCA TAA AC – 3′ and REV5′- CAA CTG CGT GCT CAT TTC CG – 3′. Human Id2: FW; 5′- TGC ATG CAC ACA CAC ACA C – 3′and REV5′- AAG AAA GGC TGG GAC CTG AG – 3′.

### Viral transduction

Production of lentiviral particles and transduction was performed as previously described [52].

### Immunohistochemistry

IHC was carried out on 4 µm thick tissue sections as described previously [55]. Detailed description of Id2 IHC scoring can be found in the supplementary methods.

### Statistics

Statistical tests were performed in Microsoft Excel and GraphPad Prism 8 using Student *t* tests assuming non-gaussian distributions. Unless stated differently, a minimum of three independent experiments, with three biological replicates, were considered. Error bars show either standard deviation (SD) or standard error of the mean (SEM), as stated in the figure legend. Western blots were quantified using ImageJ, converting pixel intensity to white/black scales and normalized to loading controls. Statistical test on the clinical tissue micro array of invasive breast cancer was performed using IBM SPSS Statistics version 27.0.0.0. Associations between categorical variables were calculated using the 369 Pearson’s Chi-square test. *P*-values <0.05 were considered statistically significant. For the bioinformatics analysis, Wilcoxon Rank tests were performed. Violin plots were generated using GraphPad Prism 8.

### Supplementary methods

Additional experiments procedures regarding anoikis resistance and FACS analysis, RNA sequencing, co-immunoprecipitations, RT-qPCR, colony formation assays, lung colonization assays, immunofluorescence microscopy, and analysis of human expression data are available in the supplementary information [56–67].

## Supplementary information

Collated Supplemental Information
