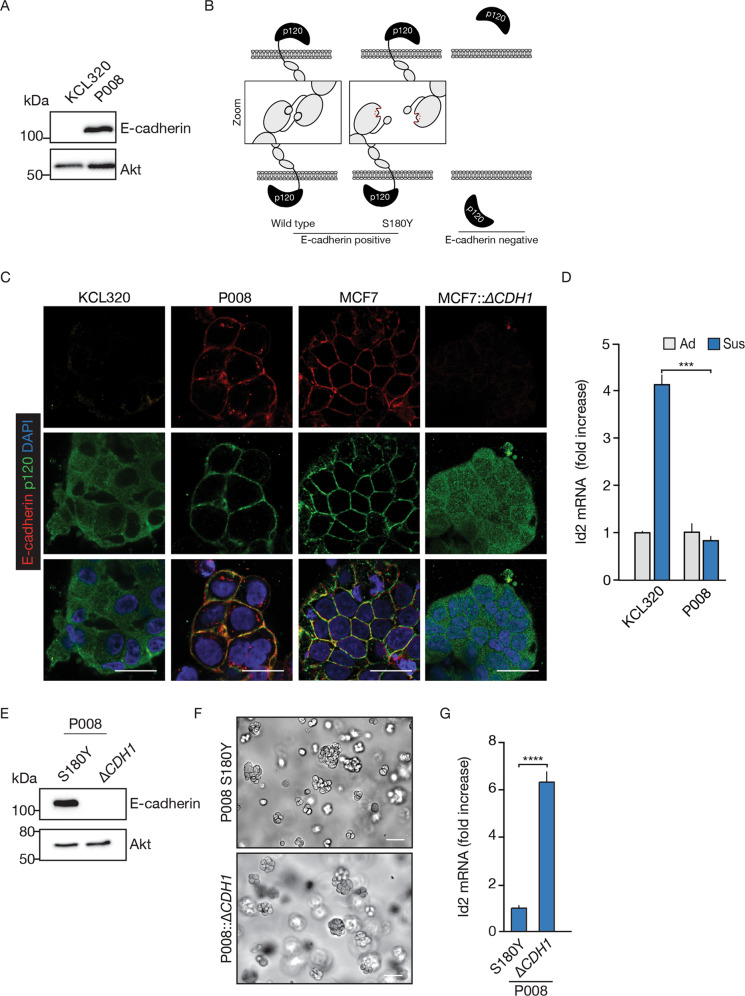# Correction: Loss of E-cadherin leads to Id2-dependent inhibition of cell cycle progression in metastatic lobular breast cancer

**DOI:** 10.1038/s41388-022-02355-1

**Published:** 2022-05-24

**Authors:** Max A. K. Rätze, Thijs Koorman, Thijmen Sijnesael, Blessing Bassey-Archibong, Robert van de Ven, Lotte Enserink, Daan Visser, Sridevi Jaksani, Ignacio Viciano, Elvira R. M. Bakker, François Richard, Andrew Tutt, Lynda O’Leary, Amanda Fitzpatrick, Pere Roca-Cusachs, Paul J. van Diest, Christine Desmedt, Juliet M. Daniel, Clare M. Isacke, Patrick W. B. Derksen

**Affiliations:** 1 Department of Pathology, University Medical Center Utrecht, Utrecht, The Netherlands; 2 Department of Biology, McMaster University, Hamilton, ON Canada; 3 Institute for Bioengineering of Catalonia (IBEC), the Barcelona Institute of Technology (BIST), Barcelona, Spain; 4 Laboratory for Translational Breast Cancer Research, Katholieke Universiteit, Leuven, Belgium; 5 The Breast Cancer Now Research Unit, King’s College London, London, United Kingdom; 6 Breast Cancer Now Toby Robins Research Centre, The Institute of Cancer Research, London, United Kingdom

**Keywords:** Breast cancer, Cadherins

Correction to: *Oncogene* 10.1038/s41388-022-02314-w, published online 18 March 2022

Following the publication of this article, the authors noted an error in Fig. 6C. The merged (bottom left) panel for the human ILC organoid KCL320, did not display the correct z-plane. The correct merged image has now been provided. The authors confirm this does not affect the conclusions of the study in any way.

The original article has been corrected.