# Supplementary material for: Loss of E-cadherin leads to Id2-dependent inhibition of cell cycle progression in metastatic lobular breast cancer
Source: Oncogene. 2022 Apr 18;41(21):2932–44. doi: 10.1038/s41388-022-02314-w (PMC9122823; doi:10.1038/s41388-022-02314-w)
Supplement: Supplementary file 1 — Collated Supplemental Information [file 41388_2022_2314_MOESM1_ESM.pdf]

Supplemental Materials:

This document contains:

- Supplemental Material and Methods
- Supplemental Figure legends 1-8
- Supplemental Table legend 1
- Supplemental Figures 1-8
- Supplemental Table 1

Supplemental Material and Methods:

Cell line culture

For anchorage-independent cultures, 100,000 or 20,000 cells were seeded in 6-well or 24-well ultra-low cluster plates (Corning) and incubated for the indicated periods. For the lentiviral E-cadherin reconstitution experiments, cells were allowed to recover for 48hrs after infection and subsequently selected for two weeks on 200μg/mL G418/Geneticin. Doxycycline was used at 2 μg/mL, and added in suspension, to induce expression plus facilitate E-cadherin reconstitution. For the generation of FUCCI-2 reporter cell lines, a population was derived through FACS single cell sorting of double positive (Geminin-AG and Cdt-KO2) cells after dual transduction with lentiviral FUCCI-2 particles. For western blot analysis, suspension cells were washed in PBS, centrifuged, and lysed using sample buffer as described [26]. For fluorescence microscopy, cells were spun onto glass slides using a Cytospin 4 cytocentrifuge (Thermo Scientific) at 600 rpm for 4 minutes.

Organoid cell culture

The KCL320 PDO model was derived as follows: Cerebrospinal fluid (CSF) was collected from a female patient with a diagnosis of leptomeningeal metastasis after informed consent as part of a non-interventional clinical trial (BTBC study REC no.: 13/LO/1248, IRAS ID 131133; Principal Investigator: Prof. Andrew Tutt; study title: “Analysis of functional immune cell stroma and malignant cell interactions in breast cancer in order to discover and develop diagnostics and therapies in breast cancer subtypes”). The P008 PDO model was generated as follows: Following informed consent (REC number 2042; Principal Investigator Prof. Clare Isacke; study title "Breast cancer invasion and metastasis") pleural fluid was collected from a 55-year old female patient diagnosed with metastatic ILC metastasis. These studies were conducted adhering to the principles of the Declaration of Helsinki. Cells isolated from the KCL320 CSF sample were plated into 3D culture in a 1:1 mix of OcellO PDX medium (OcellO B.V.) and hydrogel and overlaid with OcellO medium supplemented with 10^{-11} M 17β-estradiol, 10 ng/mL neuregulin-1, and 10 ng/mL EGF, as described previously [54–56]. Cells from the P008 pleural fluid

sample were adapted for 2D culture in vitro as described by Liu et al. (2012) and then were adapted for 3D culture in Matrigel. All cultures were maintained in humidified incubators at 37°C, 5% CO_2. Established tumor organoids were routinely tested for mycoplasma and had their identity confirmed using short tandem repeat (STR) testing using GenePrint 10 (Promega). Human ILC organoid models were resuspended in 30μL drops of cold 10mg/mL Cultrex growth factor reduced Basement Membrane Extract (BME). The cell suspension was distributed in a 24 well plate (Greiner) over three drops per well. Upon full gelation, 500μL of breast cancer (BC) organoid medium, containing Advanced DMEM/F12+++ (ADF12; ThermoFisher Scientific), 1x Glutamax, 10mM HEPES, antibiotics, 20X OcellO™ PDX additive, 10 ng/mL Heregulin (Peprotech) and 10 ng/mL Epidermal Growth Factor (Peprotech) was added, and plates were transferred to humidified 37 °C / 5% CO_2 incubators. Medium was refreshed every 2 to 3 days and organoids were passaged every 1 to 3 weeks. For passaging, organoids were resuspended in 2 mL of cold ADF12 and were dissociated by either mechanical sheering or resuspension in TrypLE Express (ThermoFisher) and incubated for 1 to 5 min at 37 °C10 mL cold ADF12 and cold centrifugation for 5 min at 1500 rpm. Organoid structures were resuspended in cold BME and reseeded in a 1:3 drop ratio, preserving high density of organoids. Both organoid lines were cultured separately to prevent cross contamination. For anchorage-independent cultures, 20,000 cells, were seeded in 6-well or 24-well ultra-low cluster plates (Corning) and incubated for the indicated periods. E-cadherin knock-out P008::Δ*CDH1* clones were generated using a lentiviral CRISPR-Cas9 CDH1 targeting vector previously used [53]. Polyclonal P008::Δ*CDH1* cultures were established by two weeks of 1μg/ml Puromycine selection. Afterwards, single organoid structures were clonally expanded and verified for E-cadherin by western blot, qPCR and TIDE-analysis (FW 5'- ATT AGC TGG GCG TGG CGA C – 3' and REV 5'- GAA GAT CGG AGG ATT ATC GTT GGT GTC AG -3') for CRISPR-Cas9 editing efficiency. For western blot analysis, suspension cells were washed in PBS, centrifuged, and lysed using sample buffer as described [26]. For fluorescence microscopy, cells were spun onto glass slides using a Cytospin 4 cytocentrifuge (Thermo Scientific) at 200 rpm for 4 minutes.

Anoikis resistance and FACS analysis

Anoikis resistance was analyzed by seeding 25,000 cells per well (in 500 μL) in a 24-well ultra-low cluster polystyrene culture dish (Corning). After 1 day and 4 days, for mouse and human cells respectively, cells were harvested and resuspended in 75 μL of Annexin V buffer supplemented with Annexin V (1:1000; IQ products) and propidium iodide (Sigma-Aldrich). The percentage of viable cells was defined as the double-negative population over the total measured cells. Anoikis resistance was defined as the double negative control cells set to 1.

mRNA expression profiling

Cells were seeded in serum-containing medium, either into a 6-well plate grown to 80% confluency or grown in suspension on ultra-low cluster plates. After washing in Ca^{2+}/Mg^{2+}-containing PBS, RNA was isolated and purified using the RNeasy kit (Qiagen), followed by DNase treatment (Qiagen). RNA library preparation was performed as previously described [59] and used for cluster generation on a HiSeq 2000 (Illumina). RPKM (Reads per kilobase million) indicates the total mRNA abundance/reads (expression of a particular gene) in a sample divided by 1 million, followed by normalization to the length and the identity of the gene. Normalization of RPKM values inherently converts the data into relative ratios, making statistical analysis unreliable. Since we observe the same trend of Id2 upregulation in three independent mILC cell lines we considered it relevant for further analysis. The resulting expression data were posted to: http://www.ncbi.nlm.nih.gov/geo/query/acc.cgi?acc=GSE81977. For the analysis of cell cycle regulatory genes (Supplementary Fig. 1) we used oligo array-based mRNA data as described in [26].

Co-immunoprecipitations

Co-immunoprecipitations were performed as previously described [26] using a rabbit Id2 antibody (1:100; #3431; clone D39E8, Cell Signaling Technology). Cells were cultured for 24 hours in anchorage-independent conditions using ultra low-cluster T75 flasks (Corning) to ensure a pure hypo-

phosphorylated Rb pool. Cytosolic and nuclear fractions were derived as described [60] and analyzed by Western blotting as previously described [26].

Reverse transcriptase quantitative PCR

Total mRNA was extracted from cell or organoid pellets using Trizol reagent (Thermo Fisher Scientific). Poly-T primers and a cDNA transcription kit (BioRad iScript Synthesis kit) were used to generate cDNA. Two PCR primer sets were used to evaluate expression values for human Id2 (set 1: FW: 5'-AGT CCC GTG AGG TCC TTA G-3' and REV: 5'-AGT CGT TCA TGT TGT ATA GCA GG-3'; set 2: FW: 5'- GCT ATA CAA CAT GAA CGA CTG CT- 3' and REV: 5'-AAT AGT GGG ATG CGA GTC CAG-3'). Primer efficiency was assessed by serial dilution. Expression values were generated using ΔΔCt values normalized to *GAPDH* and/or ACTIN for control and Id2. Experiments were performed in triplicate over three independent biological and technical settings, using the BioRad CFX96 Real-Time System and BioRad CFX manager software. For each comparison, unpaired two-tailed Student's t-tests were used to determine statistical significance.

Colony Formation Assays

Cells were seeded in triplicate at a density of 5,000 or 10,000 per well in 24 or 12 well plates. Cells were fixed with cell culture media containing 2.5% glutaraldehyde (Sigma) for 10 min, washed twice with water and stained with 0.1% Crystal Violet (Sigma) for 30 min. Cells were then washed three times with water and plates were dried overnight. Crystal violet intensity as a measure of proliferation rate was determined by washing the cells for 20 min with 10% acetic acid and measuring optical density (OD) at λ= 595 nm using a spectrophotometer. OD measurements were corrected to the day 0 control plate. Experiments were performed at least three times over three independent biological settings. All further analyses were performed in GraphPad Prism Version 8.0 for statistical significance using an unpaired two-tailed Student's t-test.

Plasmids

For the knockdown (KD) of Id2 or Rb, the following shRNA oligo nucleotides (flanked by *EcoR*I and *Age*I restriction sites) were used: mouse Id2 #1 (5'-CCG GGC TTA TGT CGA ATG ATA GCA ACT CGA GTT GCT ATC ATT CGA CAT AAG CTT TTT G-3'), mouse Id2 #2 (5'-CCG GGA CCC AGT ATT CGG TTA CTT ACT CGA GTA AGT AAC CG AAT ACT GGG TCT TTT TG -3') and mouse Rb1 (5'-CCG GGT GGA TTC TGA ACG TAC TTA ACT CGA GTT AAG TAC GTT CAG AAT CCA CTT TTT G -3'). shRNA oligonucleotides were annealed and ligated in the *EcoR*I/*Age*I-digested pLKO.1 vector (#10878; Addgene) and verified by sequencing. Plasmids expressing the FUCCI-2 reporter system (pCSII-EF-mKO2-hCdt(30/120) for G1 and pCSII-EF-mAG-hGeminin(1/1000) for S/G2/M) were a kind gift from A. Miyawaki and have been described previously [57]. The mouse *Id2* reporter plasmid (pGL4-Id2pro-1036) was a kind gift from J.R. Keller (NIH) [27]. Lentiviral vectors pLV::Id2 containing a Puromycine-selectable cassette were constructed by cloning a mouse Id2 cDNA using *Nhe*I/*Mlu*I in pLV. bc.puro [52]. For Id2 overexpression experiments OE#1: pCMV-Sport6::Id2 (Invitrogen) and OE#2 pLV::Id2 plasmids were used. The Id2-mCherry reporter plasmid was cloned by Infusion PCR from pGL4-Id2pro-136 with primers FW5'- GAT CTG CTA GCA TCG ATA GTA ACA GAA TTT TCT TTT AAA AA CAG CGA TAA GCT GT -3' and REV 5'- ATA GGT ACG CGT AAG GCT TCA TGC TCG - 3' and swapping the CMV promoter from plasmid pLV-CMV-mCherry-IRES-Puro by *Cla1/Mlu1* digestion. Inducible knockdown of Id2 in human cell lines was performed using an adapted infusion FUTG system [58], created by Gibson assembly using the backbone amplified with FW 5'-agg cta gcc tgt taa tta acc cgt gtc ggc tcc ag-3' and REV 5'-tag gga ACC GGT atc tct atc act gat agg gac tta taa gat-3' together with insert 5'- gtg ata gag atA CCG GTt ccc tag tct tct agg tct aga ctc gag acg cgt cct agg cta gcc tgt taa tta acc c-3'. Integration of shRNA was performed after digestion with *Age*I and *Xho*I followed by Infusion reaction (Takarabio) with annealed Oligo nucleotides. The human CDH1/E-cadherin Doxycycline inducible reconstitution plasmid was cloned by PCR amplification (FW 5'- GGG GAC AAG TTT GTA CAA AAA AGC AGG CTT CAC CAT GGG CCC TTG GAG CCG C-3' and REV 5'- GGG GAC CAC TTT GTA CAA GAA AGC TGG GTT TTA GTC GTC CTC GCC GCC-3', and from Gateway

BP pDONR201 recombination into pINDU20 using Gateway LR-swap to generate pINDU20::CDH1. For knockdown, the following shRNA oligo nucleotides (Flanked by infusion homologous sites) were used: human Id2 #1 (5'-TGA TAG AGA TAC CGG CCC TTC TGA GTT AAT GTC AAA TTC AAG AGA TTT GAC ATT AAC TCA GAA GGG TTT TTT CGA GAC GCG TCC TA -3') and human Id2 #2 (5'-TGA TAG AGA TAC CGG CCC ACT ATT GTC AGC CTG CAT TTC AAG AGA ATG CAG GCT GAC AAT AGT GGG TTT TTT CGA GAC GCG TCC TA -3').

Transient overexpression of Id2

Transient transfections were performed with Fugene HD Transfection Reagent according to the manufacturer's protocol (Promega). Cells were co-transfected with either the pCMV-Sport6::Id2 or pLV::Id2 vector, combined with a PGK-GFP in a DNA ratio of 1.5 Id2 vector : 0.5 PGK-GFP. Transfected cells were harvested 36 hours after transfection for anchorage-independent culture and Western blotting.

Lung colonization assays

For all mouse experiments, animals were randomized at the start. Intravenously injected cells were prepared blinded to the observer and were allocated by number instead of condition. Experiments were set up as such that cage effects can be ruled out. For metastatic lung colonization experiments, $0.5•10^6$ control or mILC-1 cells expressing luciferase and scrambled, Id2 or Rb knockdown shRNAs were intravenously injected in the tail vein of Hsd:Athymic Nude-Foxn1nu (Envigo) recipient mice. Colonization was visualized over time using a Biospace ϕ imager (Biospace) as described [62], Animals were euthanized when metastatic burden using bioluminescence exceeded $2•10^3$ photons/s/cm^2/sr. All animal experiments were according to Dutch law and approved by the Utrecht University Animal Experimental Committee (DEC-ABC no. 2012.III.12.135) under permit AVD115002015263.

Immunohistochemistry

The use of left-over material was approved by the Tissue Science Committee of the UMC Utrecht. After antigen retrieval by either boiling for 20 minutes in 10 mM citrate pH 6.0 or Tris/EDTA pH 9.0, a cooling period of 30 minutes preceded the primary antibody incubation. IHC procedures were standardized performed on the Roche Ventana™ Benchmark Stain system. Hematoxylin was used as a counterstaining. Tissue micro array scoring was done blinded to patient characteristics, and results of other IHC, by at least two observers. Protein expression and localization of Id2 was scored by estimating the percentage of positive nuclei. Intensity of cytoplasmic Id2 localization was semi-quantitatively scored as 0, 1, 2 or 3. To minimize the possibility of false-negative/positive scoring, at least 2 out of 3 tumor spots per patient had to be scored to be considered positive. Statistical analysis was performed using SPSS Statistics version 25 (SPSS Inc., Chicago, IL, USA). Associations between categorical variables were quantified using the Pearson's Chi-square test or a paired two-tailed Student's t-test. P-values <0.05 were considered statistically significant.

Immunofluorescence microscopy

Cytospun cells and organoids were air-dried for two hours, then fixed for 30 minutes using 1% paraformaldehyde. Cells that were grown adherent were washed with PBS containing Mg^{2+}/Ca^{2+} (PBS^{++}) and subsequently fixed using 1% paraformaldehyde for 30 minutes. Fixed samples were incubated with primary antibody for 1 hour at room temperature, washed with PBS^{++} and incubated with secondary antibodies for 1 hour at room temperature. Samples were stained with DAPI for 5 minutes and analyzed using a Zeiss LSM 700 (Carl Zeiss). HPF (High Power Field) represents one microscope image in high resolution. A minimal of 5 HPFs with a total of at least 120 cells in total per cell cycle state (red/green/no color) were analyzed and quantified. Images were processed using ImageJ and Adobe Photoshop CC 2018.

Analysis of human expression data

The Molecular Taxonomy of Breast Cancer International Consortium (METABRIC) dataset [64] and The Cancer Genome Atlas Consortium (TCGA) breast dataset [65], including clinical data and normalized gene expression, were retrieved through cBioportal [66] on April 8, 2020. Data from [30] and [29] were retrieved through the GEO portal [67] under their accession numbers GSE88770 and GSE68057 respectively. Subtypes were assessed according to the ER and Her2 IHC or fluorescence *in situ* hybridization status. Wilcoxon tests were performed to compare continuous to categorical variables. Associations with overall survival were performed with Cox proportional hazard regressions in univariable and multivariable approaches. In the latter, adjustments were made for age (>50 vs <=50), grade (III vs I-II), tumor size (≤2 cm, <2 cm), and nodal status (positive vs negative). Overall survival was defined from time at diagnosis to death by any cause. P-values were two-sided and statistical significance considered for $p<0.05$. All analyses were performed using R 3.5.2.

Supplemental Figure Legends:

Supplemental Figure 1: E-cadherin reconstitution in SUM44PE cells restores epithelial morphology.

Representative differential interference contrast microscopy images of two independently transduced SUM44PE::iCDH1 cell lines (inducible E-cadherin) in the absence or presence of doxycycline (DOX). Size bar = 10μm. Note the restoration of epithelial morphology following restoration of E-cadherin expression (+DOX).

Supplemental Figure 2: Depletion of Id2 induces apoptosis in anchorage-independent mILC-1 cells.

A. Western blot analysis showing Id2 expression levels upon stable expression of two independent shRNA sequences directed against Id2 in mILC-1 cells. Akt was used as a loading control. **B.** Western blots for cleaved caspase 3 on lysates from Adherent (Ad) and Suspension (Sus; Sus1=12hrs and Sus2=24hrs) cultured mILC-1 cells targeted with an Id2 (shId2) or control scrambled short hairpin (shScr). AKT was used as a loading control. **C.** RT-qPCR depicts the extent of two independent doxycycline-inducible knock-down (iKD) for Id2 in the E-cadherin mutant human SKBR3 cell line. Relative Id2 mRNA expression levels were normalized to either GAPDH or ACTB. The student's t-test was used to determine statistical significance. Error bars show the standard deviation (SD). *** = $p<0.001$.

Supplemental Figure 3: Overexpression of Id2 induces partial anoikis resistance in E-cadherin expressing breast cancer cells.

A. Western blot analysis showing Id2 overexpression in MCF7 cells from two independent CMV-driven expression vectors (OE#1 and OE#2), P-Rb S807/811 and total Rb (tRb) compared to the GFP transfection control and Akt was used as a loading controls. Protein levels were converted from pixel intensity of P-Rb S807/811 and normalized to total Rb (tRb), calculating P-Rb (Fold). **B.** FACS analysis

of apoptotic wild type MCF7 cells transfected with Mouse Id2 overexpression vectors as in (B), (OE#1 and OE#2) after anchorage-independent culturing for (sus/96hrs). Error bars show the standard deviation (SD). * = $p<0.05$, **= $p<0.01$.

Supplemental Figure 4: Cytosolic Id2 interacts with Rb in anoikis resistant cells.

A. mILC-1 cells were cultured in suspension and protein lysates were subjected to co-immunoprecipitation (IP) using monoclonal antibodies against Id2. Western blots probed for Rb (top) and Id2 (bottom) are shown. Input = 10% of the total lysates used for co-IP. **B.** mILC-1 cells were cultured in adherent or suspension conditions for 24hrs, after which cells were subjected to cellular fractionation and Western blotting. Fractionation was confirmed using Akt and Lamin A/C as cytosolic and nuclear markers respectively. **C.** E-cadherin expression levels in Trp53$^{\Delta}$, mILC-1 and Trp53 $^{\Delta}$::Δ*Cdh1* cell lines. Akt was used as a loading control. **D and E.** Id2 immunofluorescence (green)(D) Cytoplasmic Id2 expression was quantified using cytoplasmic over nuclear line scans in Trp53$^{\Delta}$, mILC-1 and Trp53$^{\Delta}$::Δ*Cdh1* cell lines (D). Note the decrease in nuclear Id2 when cells are cultured in suspension (Sus/24hrs). Size bar = 1,5μm. Representative experiments are shown from at least three independent experiments. The student's t-test was used to determine statistical significance. Error bars indicate standard deviation (SD). ns = not significant, **** = $p<0.0001$.

Supplemental Figure 5: Id2 controls anchorage-dependent growth.

A. Western blot analysis showing the extend of Id2 or Rb knockdown in mILC-1 cells. Akt was used a loading control **B and C.** Id2 is necessary for anchorage-dependent proliferation of mILC cells. Representative images of colony formation assay are shown in mILC-1 cells expressing shScr, shId2 or shRb (B). (All) Representative experiments are shown from at least three independent experiments. The student's t-test was used to determine statistical significance. Error bars show standard deviation (SD). *** = $p<0.001$.

Supplemental Figure 6: Loss of Id2 leads to DNA damage accumulation in suspension.

A - C. FUCCI-2 tagged mILC-1 cells were cultured in either adherent (Ad) or anchorage-independent (Sus/24hrs) conditions and stained for phospho-γH2AX (white; foci) using immunofluorescence. Cells in suspension were spun onto slides, fixed, stained, and analyzed (A). DNA damage foci from (A) were quantified per cell cycle condition in (B). Note the high amount of DNA damage in G2/M cells (green boxes) and the significant CDK4/6-dependent decrease of cells in the G2/M phase by the CDK4/6 inhibitor ribociclib in Id2-depleted cells (2-fold decrease). The cumulative DNA damage upon Id2 knock-down and rescue by the CDK4/6 inhibitor Ribociclib in Id2 knockdown cells is shown in (C) (All) Representative results are shown from at least three independent experiments. Significant differences are based on the number of cells in G2/M for each condition. HPF; high power field. A two-tailed Fisher's exact test was used to determine statistical significance in (B). Error bars indicate standard deviation (SD). ns = not significant, *** = $p<0.001$.

Supplemental Figure 7: The structural impact of CDH1 mutation S180Y in the ILC patient-derived tumor organoid P008.

A. A top and front view of the molecular E-cadherin strand-dimer structure model. Each monomer (red and blue) consists of two ectodomains (EC1 and EC2), at whose interface three Ca2+ ions are found (Ca-binding domain). At the right, a magnified picture of the area represented by the dashed rectangle is shown. As can be seen in this picture, the W156 of a cadherin (red) is introduced into the "acceptor pocket" of the opposite cadherin (blue) during the *trans* homophilic adhesion of two E-cadherin molecules. **B.** Representation of the main molecular interactions between the first two residues of the adhesion arm (D155 and W156) of a cadherin and the acceptor pocket of an opposite cadherin. At the left, acceptor pocket corresponding to the E-cadherin wild-type model. At the right, acceptor pocket corresponding to the S180Y mutant model. It is worth emphasizing that both structures represented in these pictures correspond to the last snapshot obtained from their respective MD simulations, after 25 ns of production run. **C.** Molecular surface representations of the wild-type model (left) and S180Y

mutant model (right) acceptor pockets. At the center, superimposition of the acceptor pockets of the wildtype (carbon atoms in gray) and S180Y mutant (carbon atoms in green) models. In all three pictures, the presence of residues S180 and Y180 are indicated.

Supplemental Figure 8: Id2 mRNA expression does not associate with prognosis in breast cancer.

Shown are hazard ratios between Id2 mRNA expression, age, tumor size, nodal status and overall survival in breast cancer patients. Univariable and multivariable approaches were considered and shown in blue and red respectively. Correlations and comparisons were quantified, and associations were computed from the publicly available TCGA, METABRIC, Michaut *et al.* 2016 and Metzger *et al.* 2013 datasets. The following four variables were binned as mentioned on the forest plot; patient age, tumor size, tumor grade and lymph node spreading. Confidence intervals were calculated using survival package in R. Statistical significance was calculated by Wald tests.

Supplemental Table legends:

Supplemental Table 1: Clinicopathological Characteristics of Breast Cancer Tissue Micro Array (TMA) samples.

Supplemental Figure 1

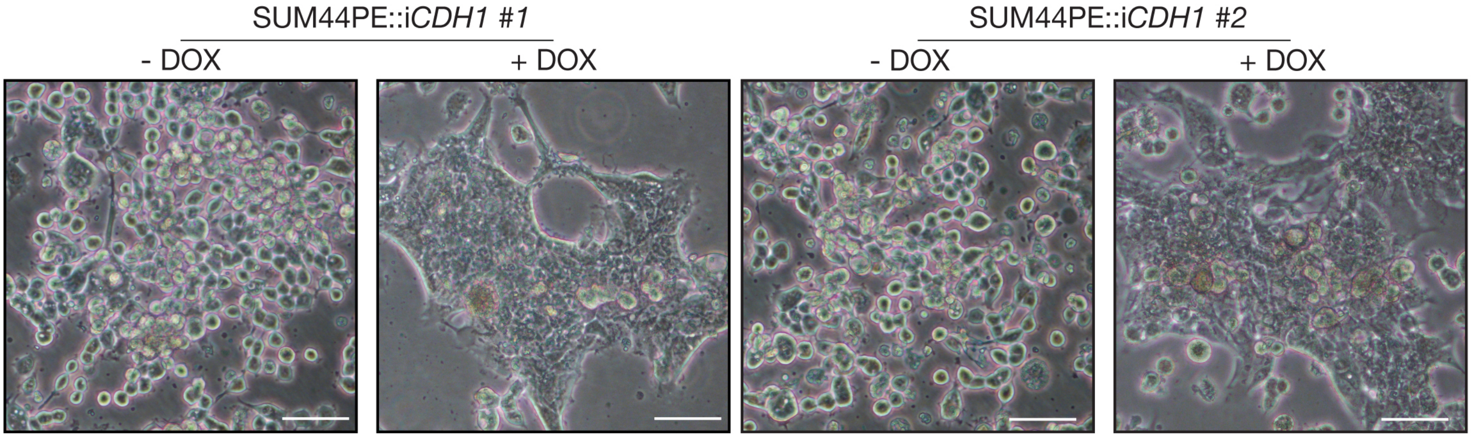

Supplemental Figure 2

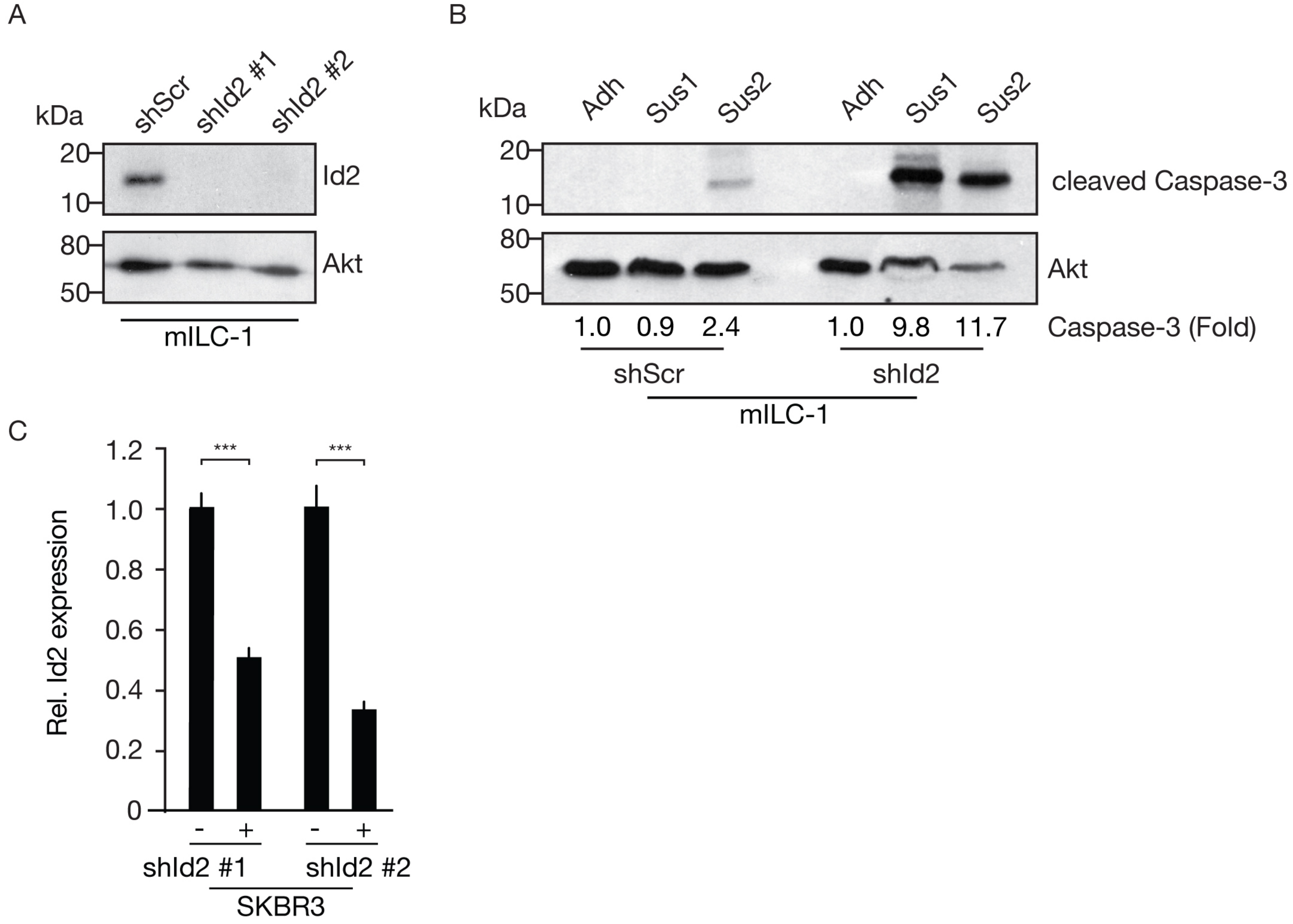

Supplemental Figure 3

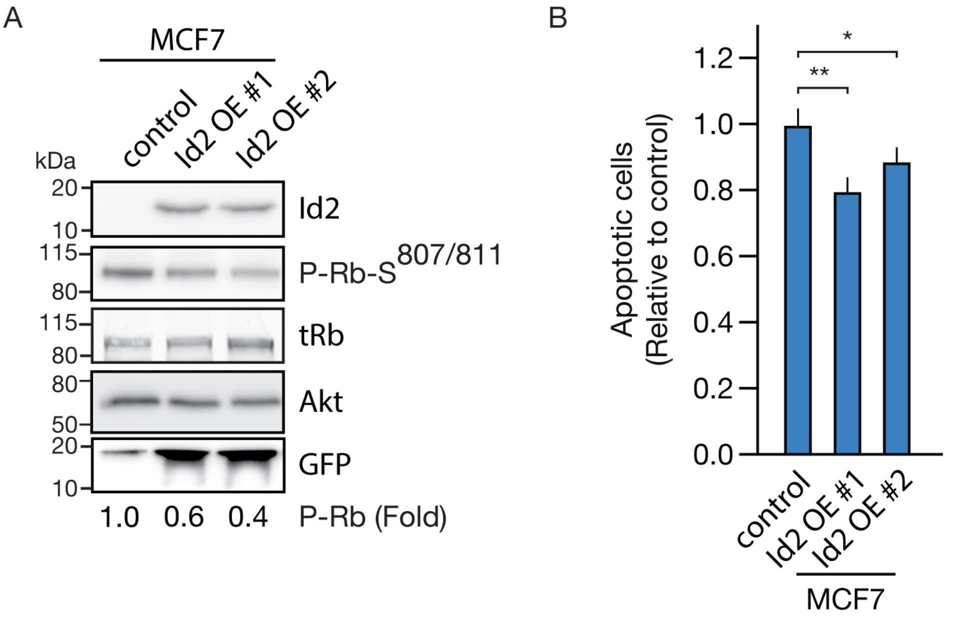

Supplemental Figure 4

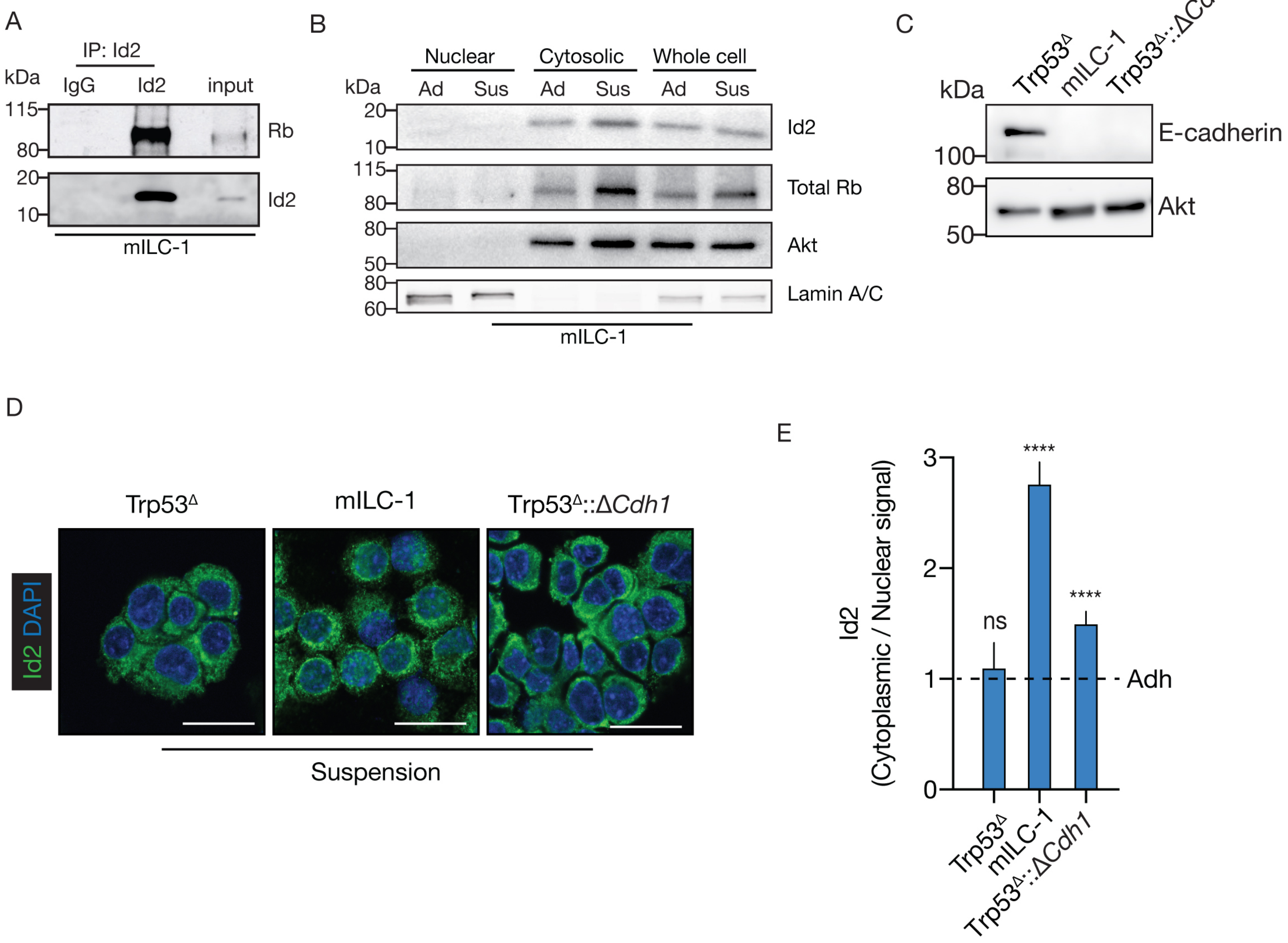

Supplemental Figure 5

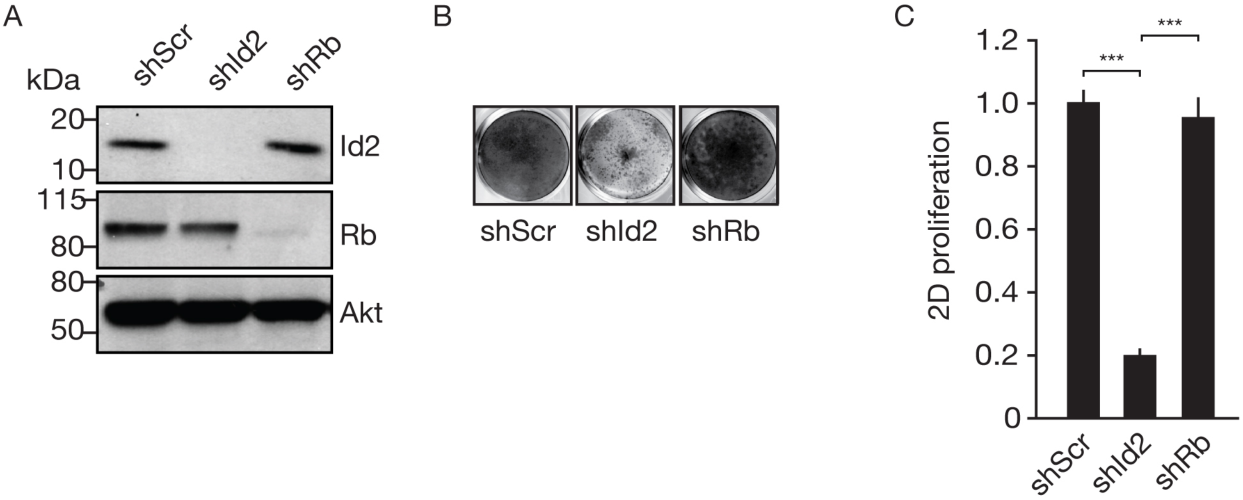

Supplemental Figure 6

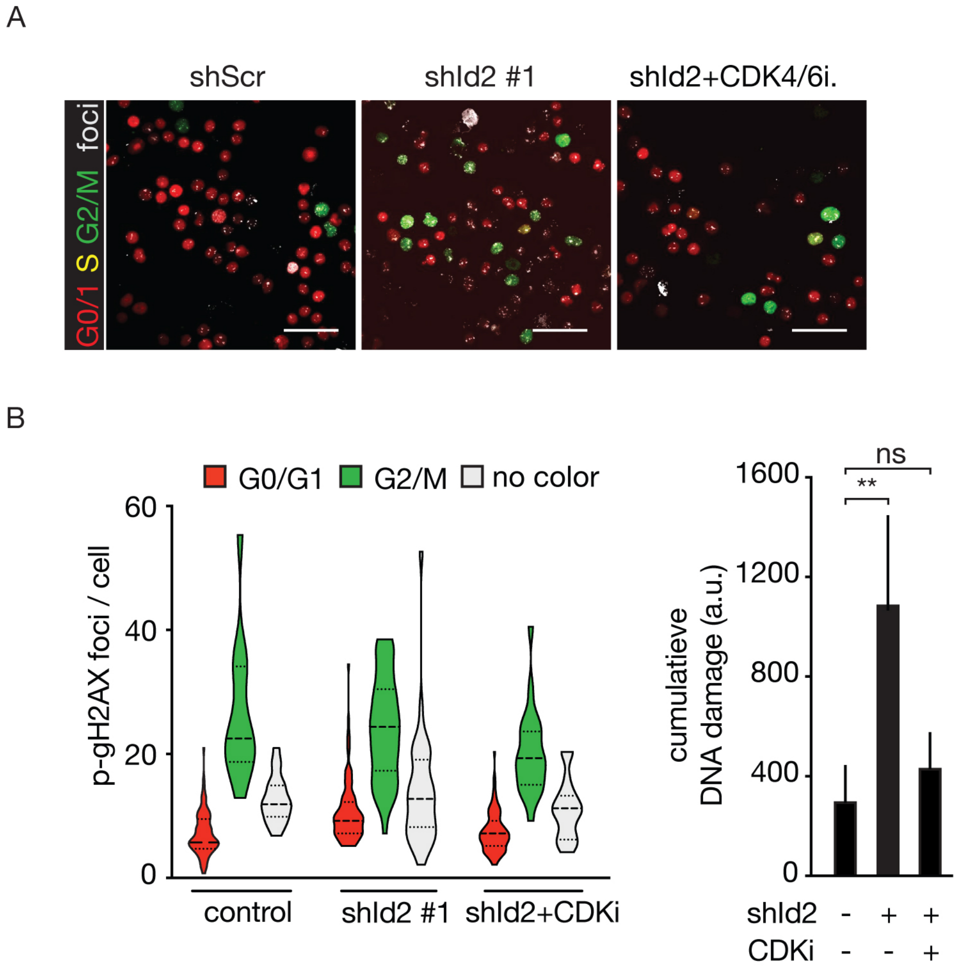

Supplemental Figure 7

A

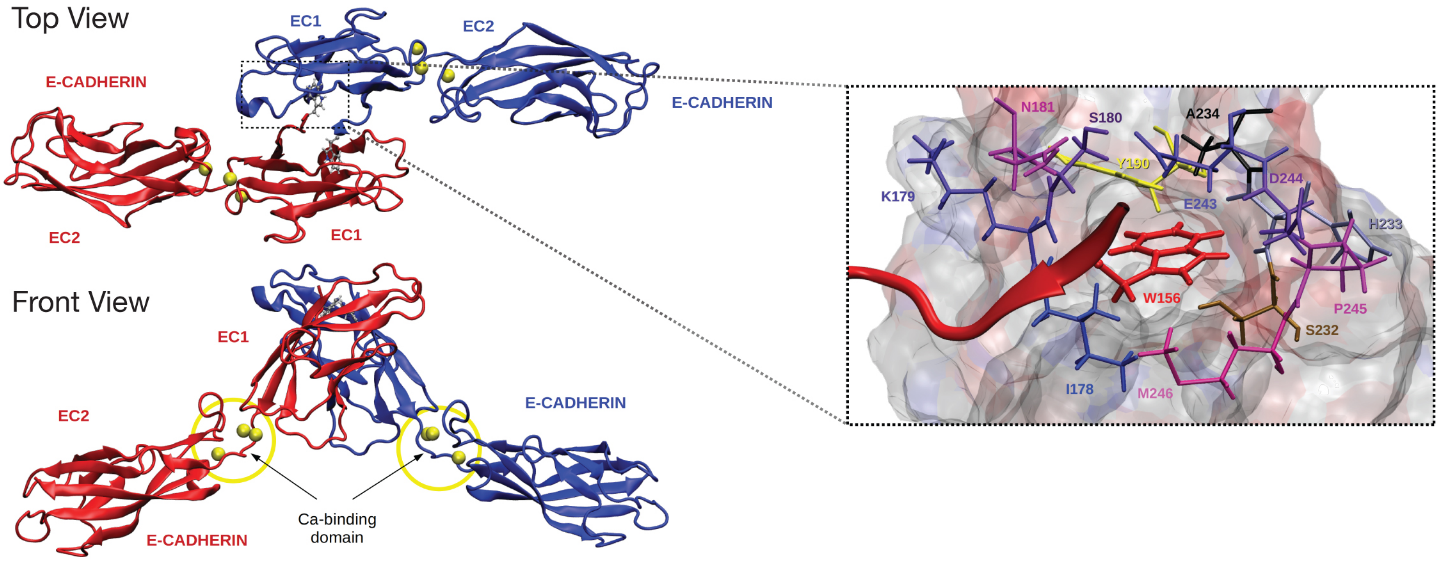

B

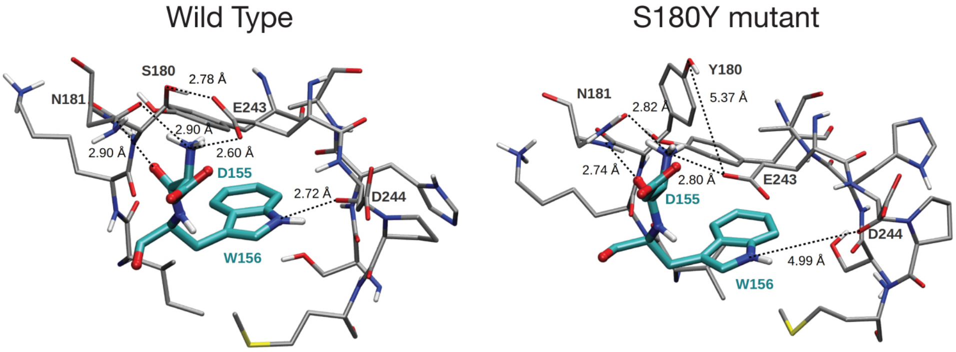

C

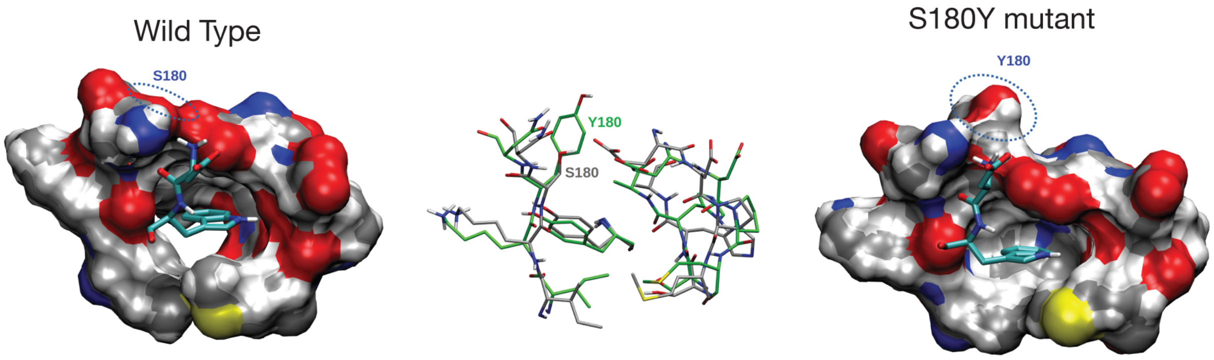

Supplemental Figure 8

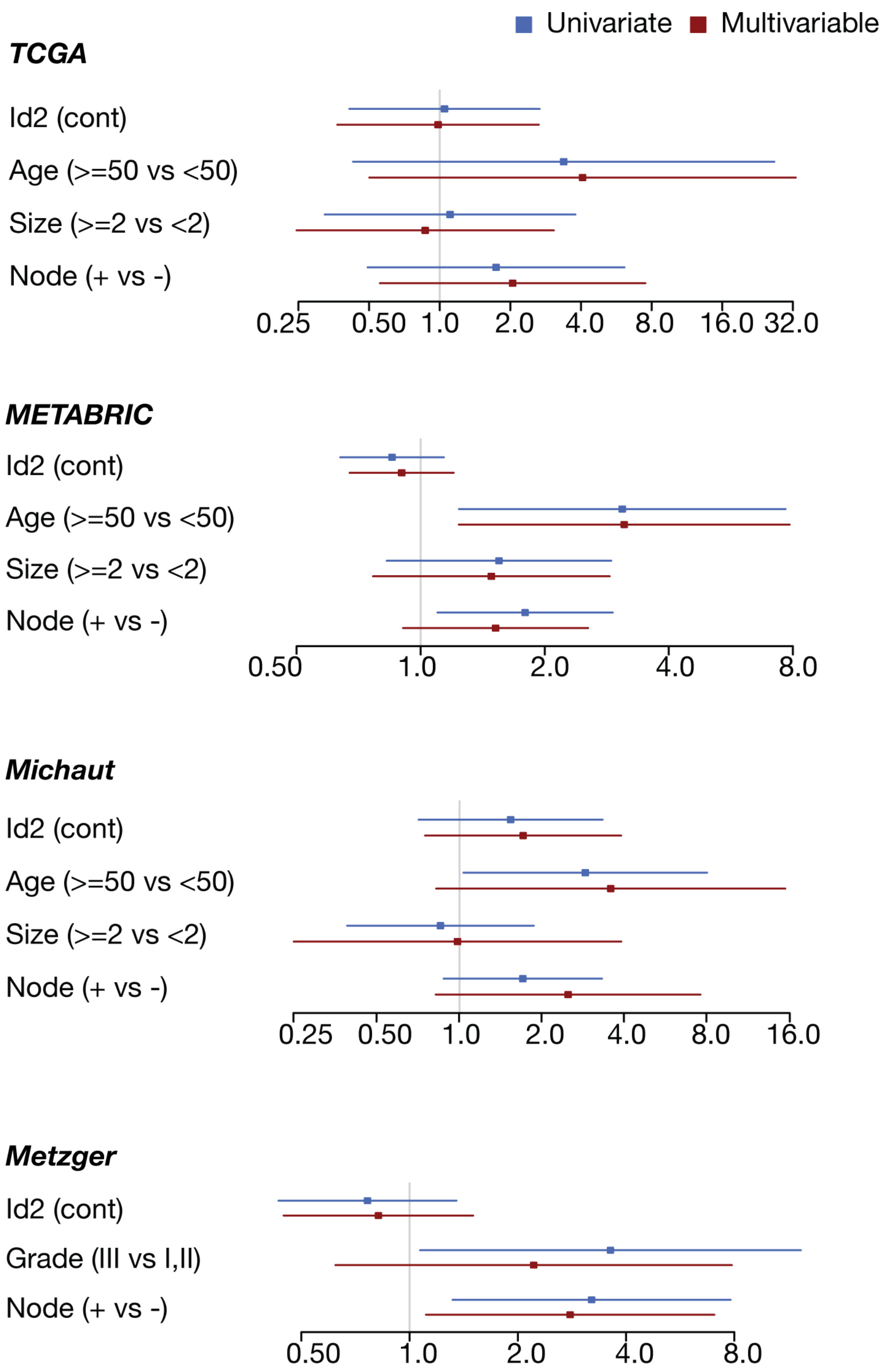

Supplemental Table 1. Clinicopathological Characteristics of Breast Cancer Tissue Micro Array (TMA) samples.

		IDC		ILC		
**Feature**	**Grouping**	**Id2 Negative**	**Id2 Positive**	**Id2 Negative**	**Id2 Positive**	
		N (%)		N (%)		p-value (chi-square)
N		81	36	12	19	
Age	Range	86	88	75	78	
		34	38	43	38	0.6667
Histological Grade	1	11 (13,6)	11 (30,6)	2 (16,7)	1 (5,3)	
	2	22 (27,2)	10 (27,8)	5 (41,7)	8 (42,1)	
	3	47 (58)	15 (41,7)	3 (25)	5 (26,3)	
	NA	1 (1,2)	0 (0)	2 (16,7)	5 (26,3)	0.0518
Lymph node status	Negative*	35 (43,2)	17 (47,2)	5 (41,7)	11 (57,9)	
	Positive**	45 (55,6)	18 (50)	5 (41,7)	3 (15,8)	
	NA	1 (1,2)	1 (2,8)	2 (16,7)	5 (26,3)	0.1224
Receptor Status	ER positive	62 (76,5)	33 (91,7)	6 (50)	8 (42,1)	0.0003
	PR positive	47 (58)	28 (77,8)	4 (33,3)	7 (36,8)	0.0066
	Her2 positive	10 (12,3)	4 (11,1)	0 (0)	0 (0)	0.2466
Tumor Size	<=	45 (55,6)	14 (38,9)	6 (50)	6 (31,6)	
	>	32 (39,5)	22 (61,1)	3 (25)	4 (21,1)	
	NA	4 (4,9)	0 (0)	3 (25)	9 (47,4)	0.2

*: negative = N0 or N0(i+); **: positive = ≥N1mi (acording to TNM 7th edition, 2010)
